# Comparative Immunogenomics of Canine Natural Killer Cells as Immunotherapy Target

**DOI:** 10.3389/fimmu.2021.670309

**Published:** 2021-09-14

**Authors:** Alicia A. Gingrich, Taylor E. Reiter, Sean J. Judge, Daniel York, Mio Yanagisawa, Aryana Razmara, Ian Sturgill, Ugur Nur Basmaci, Rachel V. Brady, Kevin Stoffel, William J. Murphy, Robert B. Rebhun, C. Titus Brown, Robert J. Canter

**Affiliations:** ^1^Department of Surgery, University of California Davis, Sacramento, CA, United States; ^2^Department of Population Health and Reproduction, University of California Davis, Davis, CA, United States; ^3^Department of Surgical and Radiological Sciences School of Veterinary Medicine, University of California Davis, Davis, CA, United States; ^4^Department of Dermatology and Internal Medicine, University of California Davis, Sacramento, CA, United States

**Keywords:** natural killer cells, comparative oncology, single cell, immunotherapy, osteosarcoma, canine

## Abstract

Natural killer (NK) cells are key effectors of the innate immune system, but major differences between human and murine NK cells have impeded translation. Outbred dogs offer an important link for studies of NK biology and immunotherapy. We analyzed gene expression of putative NK populations from healthy dogs and dogs with naturally-occurring cancers examining differential gene expression across multiple conditions, including steady-state, *in vitro* activation with cytokines and co-culture, and *in vivo* activation with inhaled IL-15 in dogs receiving IL-15 immunotherapy. We also compared dog, mouse and human CD3-NKp46+ NK cells using a novel orthologous transcriptome. Distinct transcriptional profiles between NK populations exist between conditions and *in vitro* versus *in vivo* treatments. In cross-species analysis, canine NK cells were globally more similar to human NK cells than mice. These data define canine NK cell gene expression under multiple conditions and across species, filling an important gap in translational NK studies.

## 1 Introduction

T-cell based immunotherapy has shown remarkable success in treating multiple human cancers with sometimes dramatic and durable anti-tumor effects (1). Yet, despite the clear excitement, only a fraction of patients respond to treatment, and novel strategies are needed (1). As a result, there has been a growing interest in the potential of other immune cells to meet this need, and natural killer (NK) cells are a clear candidate given their role in tumor surveillance and elimination of transformed cells without prior antigen sensitization (2, 3). This antigen-unrestricted cytotoxicity is a valuable potential asset in solid and immunologically “cold” tumors, such as osteosarcoma, where significant tumor heterogeneity exists, specific antigen targets are lacking, and response to checkpoint inhibitors has been modest (4).

Experiments from mice have been foundational to biomedical research and remain invaluable to understand mechanistic concepts necessary to propel immunotherapy into clinical practice. But intrinsic characteristics of mouse models create challenges for the translation of immunotherapy to the clinic (5). Modeling tumor latency and tumor heterogeneity which underlie host immuno-editing and tumor immune evasion mechanisms require spontaneously-arising cancers in subjects with intact immune systems. Even the evolving field of humanized mice lacks fundamental regulatory elements, such as MHC class I and II presentation and recognition, which are critical to these processes (6).

To this end, companion canines have received attention as a relevant model for studying cancer biology and therapy. Comparative oncology leverages the canine model, as dogs are outbred mammals that develop spontaneous cancers in the setting of an intact immune system (7–9). Dogs develop many of the same malignancies as humans, including osteosarcoma, lymphoma, gliomas and melanoma (9). In fact, the natural history, genetic mutations, and response to therapy of canine cancers are remarkably similar to humans (8–11).

As cancer outcomes critically depend on the interplay between genomic alterations and immune responses, few species are as well-suited for translational immunotherapy studies as companion canines (12). This is especially relevant for the study of NK cells, as significant species differences between mouse and human NK cells have hindered success. NK cells have shown clinical efficacy in blood-based cancers (13) but limited breakthroughs have occurred in solid tumors. Given the shared tumor biology, dogs are an ideal model to test NK-based immunotherapies against solid tumors. However, much less is known about canine NK cells when compared to mice and humans (14, 15). A primary reason for this is few dog-specific monoclonal antibodies are available, and this limits the ability to perform a host of techniques essential to characterize immune cells, most notably flow cytometry.

Therefore, the objective of our study was to define the transcriptome of canine NK cells using high throughput RNA sequencing (RNASeq) in both healthy dogs and dogs with cancer, including dogs receiving immunotherapy with inhaled IL-15 as part of a clinical trial. These transcriptomic studies identify pathways critical to NK effector function in resting and activated states. We also compared the transcriptome of dog NK cells to resting and activated mouse and human NK cells using a novel orthologous transcriptome comparing the expression of over 7000 conserved genes. Overall, these innovative studies offer detailed characterization of the dog NK transcriptome for the first time and shed light on the similarities and differences of dog NK cells to those of mice and humans, highlighting how dog NK cells may be leveraged for therapeutic benefit.

## 2 Results

### 2.1 Activated Canine NK Cells Expanded From a CD5-Depleted Starting Population Express Canonical NK Markers Similar to Activated CD3-CD56+ Human NK Cells

The CD5^dim^ population represents a putative marker for canine NK cells based on multiple independent studies, but questions remain as to the sensitivity and specificity of this marker given the lack of orthologue in human and mouse NK cells (16–18). Moreover, this designation has been principally based on the description of cytotoxic and cytokine-secreting functions after exposure to highly activating conditions such as IL-2, IL-15 or co-culture with K562.clone 9 feeder cells rather than functional assessment in the resting state or detailed phenotypic assessment (17, 18). Therefore, we began by performing differential gene expression (DGE) to compare dog and human NK cells from healthy donors using CD5 depletion to enrich for CD5^dim^ dog NK cells and magnetic bead separation to isolate CD3-CD56^bright^ human NK cells. We compared DGE from both the steady-state (“resting”) and after exposure to co-culture with K562 human feeder cells transduced with 4-1BBL (CD137L) and membrane-bound rh-IL21 and IL-2 for 14 days (“activated”) (19).

**Figure 1:**
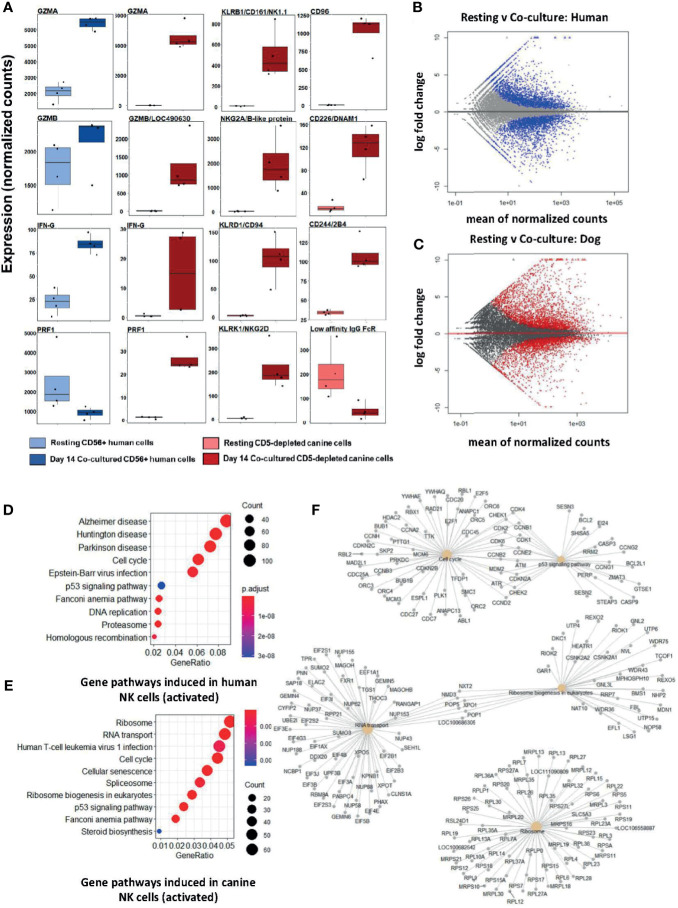
Activated canine NK cells expanded from a CD5-depleted starting population expresses canonical NK markers similar to activated CD3-CD56+ human NK cells. NK cells were isolated *via* magnetic separation for canine (CD5-depleted) or by RosetteSep NK enrichment for human NK cells (CD3-CD56+). Differential gene expression between resting canine and human NK cells compared to cells exposure to irradiated K562 human feeder cells transduced with 4-1BBL (CD137L) and membrane-bound rh-IL21 (K562C9IL21) supplemented with 100 IU/mL rh-IL2 for 14 days reveals similar profiles. **(A)** DGE using DESeq2 showed comparable expression levels of *GZMA* and *IFNG* in human and canine cells. Locus 490630 on the CanFam3.1 transcriptome was identified as *GZMB* homolog and its expression is similar in humans and canines. Resting humanCD3-CD56+ cells express more *PRF1* mRNA than resting canine CD5^dim/-^ cells. A panel of NK-associated genes demonstrated elevation in the activated state for canine CD5^dim^ cells, with the exception of the canine low affinity IgG Fc Receptor (CD16). Using the CanFam3.1 transcriptome, Locus 486692 and Locus 478984 were identified for the first time as NKG2A/B and CD16 homologs, respectively. **(B, C)** MA (ratio intensity) plot, mean of normalized counts by log fold change, compares differentially expressed genes (blue = human, red = dog) between treatment conditions. In total, **(B)** contains 3305 induced genes and 2872 repressed genes and **(C)** contains 2573 induced genes and 2448 repressed genes. Each species has a similar number of differentially expressed genes. **(D, E)** Dotplots of gene ontology analysis results (using KEGG) comparing gene pathways induced in humans **(D)** and canines **(E)**, respectively. Both populations express pathways associated with high cell turnover (eg: cell cycle, RNA transport, DNA replications) and dysfunction (eg: cellular senescence, disease-annotated). With KEGG, disease-annotated pathways denote dysfunction rather than actual pathologic state. **(F)** A more detailed representation of GO analysis as a cnetplot for activated canine CD5-depleted cells which includes specific gene names and overlap between interacting pathways. Predominant pathways include genes involved in cell cycle, p53 signaling, RNA transport, and ribosomes. Cell cycle and p53 pathways share the greatest number of induced genes.

Following feeder line co-culture, the transcriptomes of activated canine and human cells demonstrated induced expression of multiple canonical NK genes, including genes for cytotoxicity, cytokine expression, and a panel of genes from the NK Gene Complex (NKC) (**Figure 1A**). The NKC governs MHC-I receptors and killer cell lectin like receptor (KLR) genes and is highly conserved between dog and human (20, 21). Elevations of classic cytotoxicity markers *GZMA, GZMB, IFNG*, and *PRF1* were also observed in activated canine NK cells. We noted decreased expression of the IgG Fc receptor III (CD16) after co-culture activation, consistent with the human CD56^bright^CD16^dim/-^ phenotype, known for cytotoxicity. There were thousands of differentially expressed genes between resting and activated states in both species, demonstrating responses of comparable magnitude (**Figures 1B, C**). Differentially expressed genes were enriched in pathways involved in high proliferation as well as several dysfunction/disease-associated and senescence pathways in activated NK populations from both species (**Figures 1D, E**). It is important to note with KEGG, disease-annotated pathways denote dysfunction, not solely pathologic state. A closer look at the top 5 pathways for day 14 feeder line activated CD5-depleted cells in dogs is depicted in **Figure 1F**. Based on these data, activated NK cells from humans and canines bear remarkable similarity when simulated under specific co-culture conditions, with both yielding populations with high transcription of genes underlying cytotoxicity and NK activation.

### 2.2 Resting CD5-Depleted and CD3-NKp46+ Cell Populations Are Disparate but Converge on Identical Gene Expression in the Activated State

Two widely accepted phenotypic surface markers for canine NK cells are CD5^dim^ and CD3-NKp46+ (16, 22, 23). Numerous studies have correlated CD5^dim^ expression as indicative of a dog NK starting population, but in other species CD5 is classically a T cell marker, and some studies have observed CD3zeta expression in CD5^dim^ canine NK-like cells (17). As a result, the validity of using CD5^dim^ as an NK marker in dogs is unresolved. Conversely, NKp46/NCR1 is a member of the Natural Cytotoxicity Triggering Receptor (NCR) family and considered a pan-mammalian NK cell marker (20, 22, 23). In canines, the use of NKp46 as a phenotypic marker appears reliable in the identification of a smaller homogenous population (1-2.5% of PBMCs) in the resting state (22, 23). Given the potential for CD5^dim^ and NKp46 to identify heterogeneous subsets, we next examined these putative NK populations for overlap in DGE in resting and activated states.

**Figure 2:**
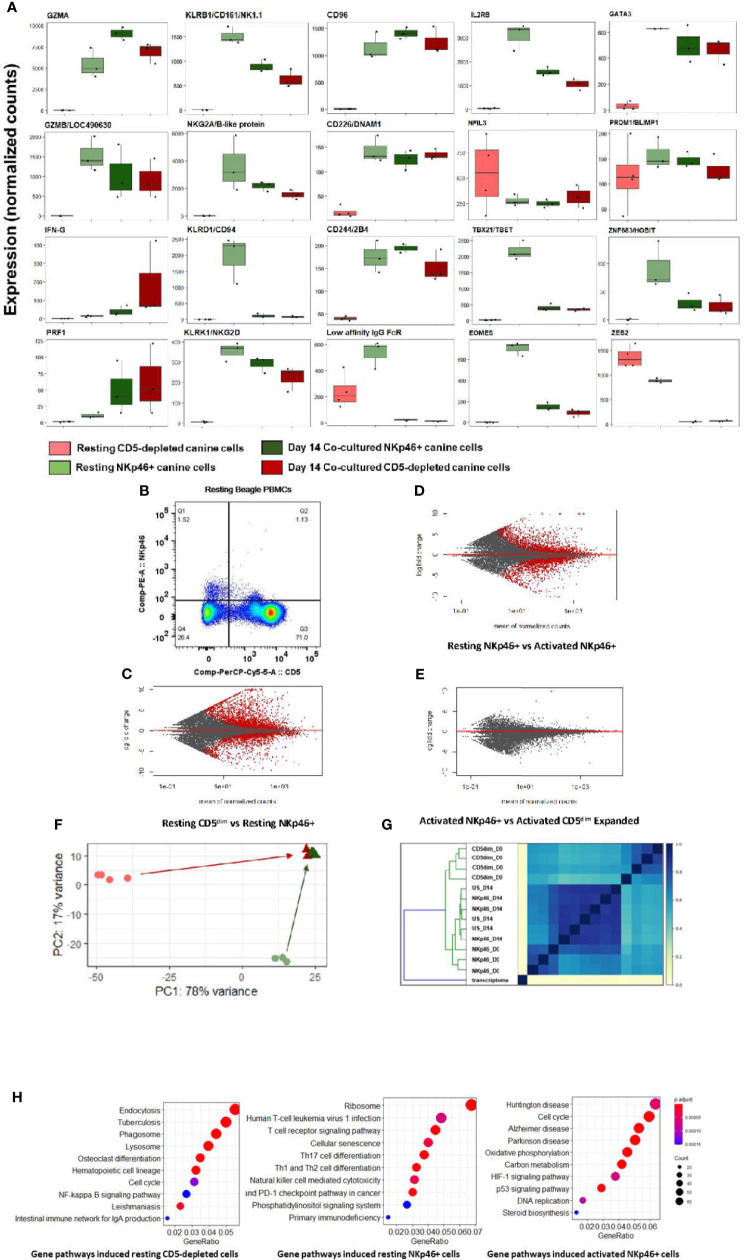
Canine CD5-depleted and sorted CD3-NKp46+ cell populations converge on identical gene expression in the activated state. Differential gene expression analysis of resting (Day 0) CD5-depleted and FACS-isolated CD3-NKp46+ versus activated (Day 14) NKp46+ and expanded CD5-depleted cells as candidate canine NK cell populations. **(A)** Individual gene counts of canonical NK markers by treatment group: CD5-depleted resting, CD3-NKp46+ resting, CD3-NKp46+ activated and unsorted expanded CD5-depleted activated cells. Sorting on CD5-depleted in the activated state is not possible as expression is lost after 14-days of feeder line co-culture. Resting CD3-NKp46+ cells have higher expression of canonical NK-associated genes than CD5-depleted cellsand therefore may represent a subset of circulating NK cells. **(B)** Flow cytometry results for CD5dim versus NKp46 staining of the starting population from healthy beagle PBMCs, demonstrating overlapping of the two phenotypes. **(C, D, E)** MA (ratio intensity) plot, mean of normalized counts by log fold change, compares differentially expressed genes (in red) between treatment conditions. In total, Figure 2B contains 2888 induced genes and 2109 repressed genes, Figure 2C contains 1893 induced genes and 1896 repressed genes, and Figure 2D contains just 1 induced gene and 7 repressed genes which are differentially expressed between the cell populations. Thectivated CD3-NKp46+ cells compared to activated unsorted cells in Figure 2D have very few differentially expressed genes and thus nearly identical transcriptional profiles. **(F)** Principal component analysis reveals resting CD5-depleted cells drive variance for PC1, while resting CD3-NKp46+ cells drive variance for PC2 **(F)**. Almost no variance between activated CD3-(E). **(G)** The similarity matrix demonstrates Jaccard index (J(A,B) = |A∩B| / |AUB|) approaching 1 (identical) between activated CD3-NKp46+ and CD5-depleted expanded cells based on pre-abundance count hash sketches. This signifies similarity based on fastq sequences prior to alignment to a transcriptome or normalization of counts. (Key: CD5dim_D0 = resting CD5dim cells, NKP46_D0 = resting NKp46+ cells, NKp46_D14 = coculture activated NKp46+ cells, US_D14 = unsorted coculture activated cells expanded from a CD5dim resting population.) **(H)** Dotplots of gene ontology analysis results (using KEGG) comparing gene pathways induced in resting CD5-depleted cells, resting CD3-NKp46+ cells and activated CD3-NKp46+ cells. Note that pathways induced in CD5-depleted cells include phagocytosis, endocytosis and lysosome functions which have been broadly implicated in innate immune cell pathways including NK cell trogocytosis. Also note resting CD3-NKp46+ cells express the NK cell-mediated cytotoxicity pathway, but also pathways associated with T-cell development. As before, activated CD3-NKp46+ cells express pathways associated with pathways associated with both high replication and cellular dysfunction.

Notably, at steady-state, the CD5-depleted and flow-sorted CD3-NKp46+ populations demonstrated distinct transcriptional profiles (**Figure 2**). For example, resting CD3-NKp46+ cells showed high expression of NKC genes in the absence of stimulation, including *KLRB1/*NK1.1 and *KLRK1*/NKG2D. Classic NK cell genes *CD96, CD226, 24B* and *GZMB* were also elevated in CD3-NKp46+ cells at rest (**Figure 2A**). However, in resting CD5-depleted cells, these genes had much lower expression (aside from CD16, which was higher at baseline than the NKp46+ subset). Resting NKp46+ putative dog NK cells also expressed higher levels of transcription factors and receptors classically associated with NK development and maturation, including IL2RB, GATA3, and HOBIT, among others (**Figure 2B**). Taken together, these data demonstrate that in steady-state, CD3-NKp46+ cells have greater induction of genes classically associated with NK cell phenotype and function, whereas CD5-depleted cells appear to manifest a lower baseline expression of NK activation and cytotoxicity markers. These data suggest that CD5^dim^ expression among resting, non-stimulated dog PBMCs has limited sensitivity and specificity (especially compared to NKp46) in identifying dog NK cells, although the potential for species differences in NK maturation and development as well as differences in marker expression to contribute to these results remain possible (24). Importantly, flow cytometry provides evidence of two distinct resting NKp46+ populations in healthy beagle PBMCs, including one which is CD5- and one which is CD5^dim^, but none which are CD5bright (**Figure 2B**).

Remarkably, despite differences in the CD5-depleted and CD3-NKp46+ subsets at rest, the transcriptional profiles of these two populations converged to a nearly identical state after 14 days of co-culture, with few (< 1%) differentially expressed genes between the activated CD3-NKp46+ or CD5^dim^ expanded populations based on FACS sorting (**Figures 2C–E**). This high similarity is seen after calculation of Jaccard similarity and principal component analysis (PCA) (**Figures 2F, G**). These findings suggest that each population contains NK cells that are selected for rapid and dominant growth under co-culture conditions and converge on an identical transcriptional profile.

Using GO analysis, both resting cell populations possess transcriptional evidence consistent with NK cells (**Figure 2H**), but also were enriched for pathways accounting for either broader innate or T cell functions. Resting CD5-depleted cells had a higher expression of gene pathways associated with phagocytosis and endocytosis, suggesting the presence of non-NK cells in the resting population although this is likely a consequence of the CD5 depletion technique which, by negative selection, eliminates CD5bright cells and enriches for CD5 dim cells, but also retains the CD5 negative cells which are highly likely to bias the DGE results toward non-NK pathways and genes. In contrast, resting CD3-NKp46+ cells had higher expression of mixed NK and T cell pathways, to include NK cell mediated cytotoxicity and T-cell differentiation (**Figure 2H** and **Supplemental Figures 1, 2**). Taken together, the expression of NKC genes, global transcriptional profiles and GO analysis are consistent with strong features of NK cells in the CD3-NKp46+ subset, while raising questions about the resting CD5 depleted subset which may related to the presence of other immune cells in the CD5 negative populations.

### 2.3 Sorted CD5^dim^ Cells Are More Homogeneous Compared to CD5-Depleted Cells but Retain Distinct Gene Expression When Compared to NKp46+ Cells in Steady-State

A well-accepted method to enrich for canine CD5^dim^ cells is *via* magnetic separation and negative selection, which depletes CD5^bright^ cells but retains CD5- cells, as noted above. When co-culture is performed, these non-NK cellular constituents appear to be selected out given the highly tailored culture techniques to promote NK activation and expansion. Acknowledging that the presence of CD5- cells will bias gene expression of an aggregate population in bulk RNA sequencing, we next elected to compare DGE between canine cells isolated using standard CD5-depletion *via* magnetic separation to a purified CD5^dim^ population isolated by FACS-sorting.

**Figure 3:**
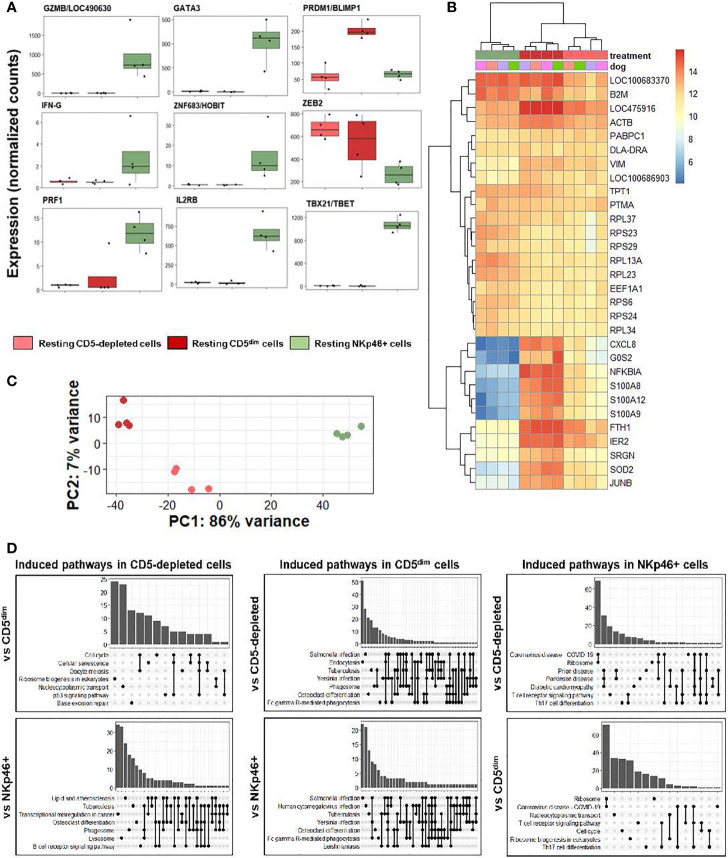
CD5-depleted and CD5^dim^ cells retain gene expression related to broad innate immune cell function when compared to NKp46+ cells in steady-state. DGE was performed between canine cells isolated using CD5-depletion *via* magnetic separation, cells isolated *via* FACS for CD5^dim^ and cells isolated *via* FACS for NKp46+ in the steady-state (at rest). The purpose of this experiment was to compare the gene expression between three most common starting populations described most frequently in the canine NK cell literature. **(A)** Individual normalized gene counts of canonical NK markers by treatment group. Within these classically NK-associated genes, CD5-depleted and CD5^dim^ expression is similar and much lower than expression in NKp46+ cells, with the exception of PRMD1/BLIMP1 and ZEB2 which is higher in the CD5-depleted and CD5^dim^ populations. **(B)** A heatmap of the top 30 most differentially expressed genes demonstrates a subset of genes induced in CD5^dim^ cells, compared to the other two populations, none of which are classically associated with NK cell function. CXCL8 is the gene for interleukin-8 and most commonly secreted by macrophages. The G0S2 gene governs the G0/G1 switch and governs cell cycle progression in blood mononuclear cells. FTH1 encodes ferritin heavy chain associated with iron binding. **(C)** Principal component analysis demonstrates the variance between CD5^dim^ and NKp46+ cells drives PC1 (86%), whereas the difference between CD5-depleted and CD5^dim^ cells drives the variance for PC2 (7%), which is much smaller. **(D)** Gene-ontology (GO) analysis between the cell populations is depicted as UpSet plots. UpSet plots the intersections of a set as a matrix. In this case, each set on the left represents a gene pathway. The quantity in the bar graph represents the normalized number of counts for that gene pathway. When genes contribute to multiple gene pathways (for example, cell cycle and oocyte meiosis) a bar connects the dots in the matrix. Thus, performing GO analysis the cell pathways on the left of each graph denote the pathways induced in a specific population when compared to a second. Gene overlap between pathways depicted by connected dots. In this case, CD5-depleted and CD5^dim^ cells share induced pathways related to broad innate functions, to include phagosome, lysosome and endocytosis. In contrast, NKp46+ cells appear to have induced pathways involved in T cell function when compared to CD5-depleted and CD5^dim^ cells. None of the populations appear to possess induced pathways related to NK cell function when compared to the others. It is important to note this does not denote the absence of NK cell function in the populations, but rather the absence of significant *differences* in any NK cell functions between the three populations.

We first analyzed individual normalized gene counts of canonical NK markers among resting CD5-depleted, sorted CD5dim, and sorted NKp46+ obtained from healthy beagle PBMCs (**Figure 3A**). Within these classically NK-associated genes, CD5-depleted and CD5^dim^ expression showed similar gene expression which was notably much lower than expression of these canonical NK genes by NKp46+ cells, with the exception of PRMD1/BLIMP1 and ZEB2 which were higher in the CD5-depleted and CD5^dim^ populations. PRMD1/BLIMP1 has been shown to be a negative regulator of NK function in human and mouse studies, often induced in response to activation and decreases sensitivity to IL-2 (25). ZEB2 has been linked to NK cell maturation (26). Importantly, however, BLIMP1 and ZEB2 are not NK specific genes, and expression has been observed in diverse immune populations, including B cells. Therefore, although sorted CD5^dim^ cells show a more uniform DGE profile than CD5 depleted dog immune cells, these data further reinforce the conclusion that CD5^dim^ is a non-specific marker which is likely of limited utility for positively identifying dog NK cells.

We then analyzed qualitative gene expression across these subsets, as depicted in a heatmap of the top 30 most differentially expressed genes (**Figure 3B** **).** This demonstrates a subset of genes induced in CD5^dim^ cells compared to the other two populations, none of which are classically associated with NK cell function. CXCL8 is the gene for interleukin-8 and most commonly secreted by macrophages. The G0S2 gene governs the G0/G1 switch and governs cell cycle progression in blood mononuclear cells. FTH1 encodes ferritin heavy chain associated with iron binding. Genes coding for proteins in the S100 family also seem to be induced in CD5^dim^ cells, less so in CD5-depleted cells and actively repressed in NKp46+ cells. Classically, S100 proteins contribute to inflammation and can be secreted by a variety of immune cells (27).

Principal component analysis (**Figure 3C**) demonstrates the variance between sorted CD5^dim^ and NKp46+ cells which drives PC1 (86%), whereas the difference between CD5-depleted and CD5^dim^ cells drives the variance for PC2 (7%), which is significantly smaller. Thus, the CD5-depleted and CD5^dim^ populations have a much higher degree of similarity to each other than either population shares with NKp46+ cells despite the presence of a small component of CD5^dim^ cells which express NKp46.

Gene-ontology (GO) analysis between the cell populations is depicted as UpSet plots (**Figure 3D**). Here, gene pathways with shared gene counts are represented in the matrix. As before, CD5-depleted and CD5^dim^ cells share induced pathways related to broad innate functions, such as phagosome, lysosome and endocytosis, which are not classically associated with NK phenotype or function. CD5-depleted cells show induced genes in the B-cell receptor signaling pathway, which is in keeping with the known fact that this population contains CD5- cells, such as B cells. CD5^dim^ cells have induced genes in the Fc gamma R-mediated phagocytosis pathway, often seen in dendritic cells (28). In contrast, NKp46+ cells appear to have induced pathways involved in T cell function when compared to CD5-depleted and CD5^dim^ cells, although there is known overlap between cytotoxic T and NK gene pathways.

Taken together, these data provide clear evidence that of the three populations, NKp46+ cells demonstrate gene expression that, in the steady state, most closely corresponds with classical NK cell function. The CD5-depleted and CD5^dim^ populations are quite similar and both populations demonstrate a more heterogeneous pattern of gene expression encompassing many innate immune cell functions.

### 2.4 Single-Cell Analysis of Resting CD5^dim^ and CD3-NKp46+ Canine Cells Confirms Greater Heterogeneity in CD5^dim^ Compared to the NKp46+ Population

Based on our findings, we then sought to confirm at single cell resolution that CD5^dim^ expression identifies a heterogeneous population which includes non-NK cell innate lymphocytes, whereas NKp46 is a more specific marker to define canine NK cells, but may not identify all resting NK cells in the steady-state. To further explore the overlap between these two populations, we performed single-cell (sc)RNASeq to examine gene expression within individually FACS-sorted CD3-NKp46+ and CD5^dim^ cells from healthy donors in both resting and co-culture activated states.

**Figure 4:**
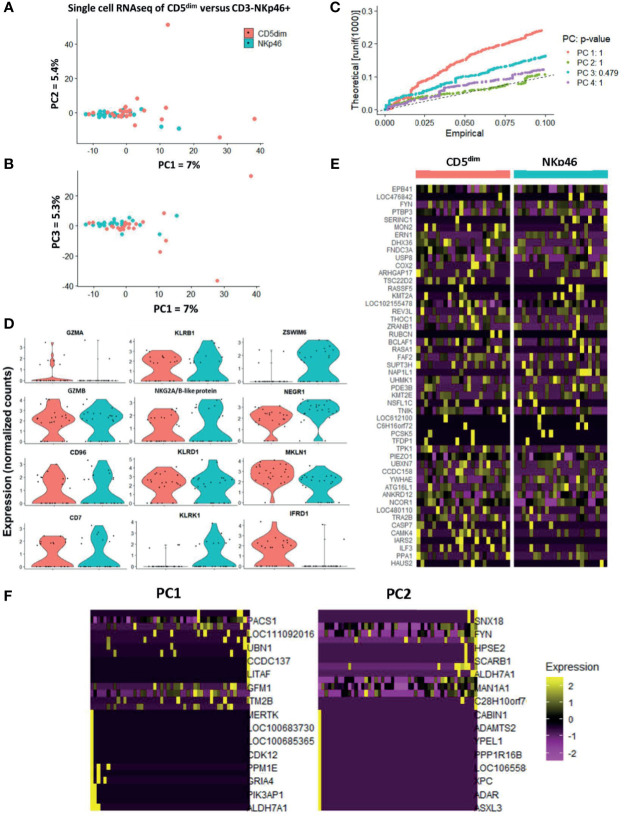
Single-cell analysis of resting CD5dim and CD3-NKp46+ canine cells confirms greater heterogeneity in CD5dim compared to the NKp46+ population. DGE following single-cell RNA sequencing of FACS-isolated resting CD5dim and CD3-NKp46+ cells from healthy beagle PBMCs. ScRNASeq was performed using the plate-based 3’ UPX Transcriptome kit with 24 cells/group given the comparative rarity of the CD3-NKp46+ group in peripheral blood. **(A–C)** PC analysis demonstrates overlap of the two populations based on their transcriptional signatures, indicating that the two populations are not unique. Variance in PCA is driven by outliers from the CD5dim population, indicating that this population is more heterogeneous and likely includes either additional subsets of NK cells or non-NK cells. Given the low percentage of variance seen in PC1, PC2 and PC3, p-values of the variance for each principal component were calculated and depicted in the associated Jackstraw plot **(C)**. The p-value for each eigenvalue does not reach significance, and thus the populations are quantitatively transcriptionally similar. **(D)** Panel of genes differentially expressed between CD5dim and CD3-NKp46+ cells with the lowest p-values in the steady-state (resting). Note the genes in the left and middle columns pertain to the NKC gene complex and Granzyme A and B, and expression is similar, save for KLRK1/NKG2D. The non-NKC genes in the right column are driving the variance for outlying CD5dim cells. **(E)** Heatmap depicting qualitative gene expression changes between the two populations, for which no consistent pattern of variation is seen between top differentially expressed genes. **(F)** Heatmap depicting genes driving the variance for PC1 and PC2, representing how the gene expression of the outlying CD5dim cells differ from the main cluster. Outlying cells have decreased expression of cell cycle genes MERTK, CDK12, PIK3AP1, among others.

PCA demonstrated the significant transcriptional overlap between individual sorted CD5^dim^ and CD3-NKp46+ cells in the blood, with outlying cells from the CD5^dim^ population driving the variance on PC1, PC2, and PC3 (**Figures 4A, B**). Interestingly, the majority of single cells between the two resting/flow-sorted cell populations was highly similar, suggesting that immune cells in the resting state can have concordant DGE, but as predicted, the CD5^dim^ population showed more heterogeneity. Given the low percentage of variance attributed to each principal component, we calculated the p-value of each eigenvalue in the corresponding Jackstraw plots (**Figure 4C**), demonstrating that the variance did not reach statistical significance. Taken together, these single cell sequencing data provide evidence in favor of the FACS-isolated CD5^dim^ and NKp46+ populations as being quantitatively similar in differential gene expression. Analysis of NKC genes revealed significant difference in only KLRK1/NKG2D between the resting CD5^dim^ and NKp46+ populations (**Figure 4D**). Gene expression driving the variance for outlying CD5^dim^ cells was related to non-NKC gene expression, including MERTK, CDK12, PPM1E, GRIA4, PIK3AP1 and ALDH7A1 (**Figures 4E, F**). Therefore, these data further reinforce that CD5^dim^ appears to be a less specific marker for NK cells and contains gene expression less specific for NK cells.

### 2.5 Single-Cell Analysis of Activated CD5^dim^ and CD3-NKp46+ Canine NK Cells Shows Heterogeneity in Time-to-Response for NK Cell Activation

**Figure 5:**
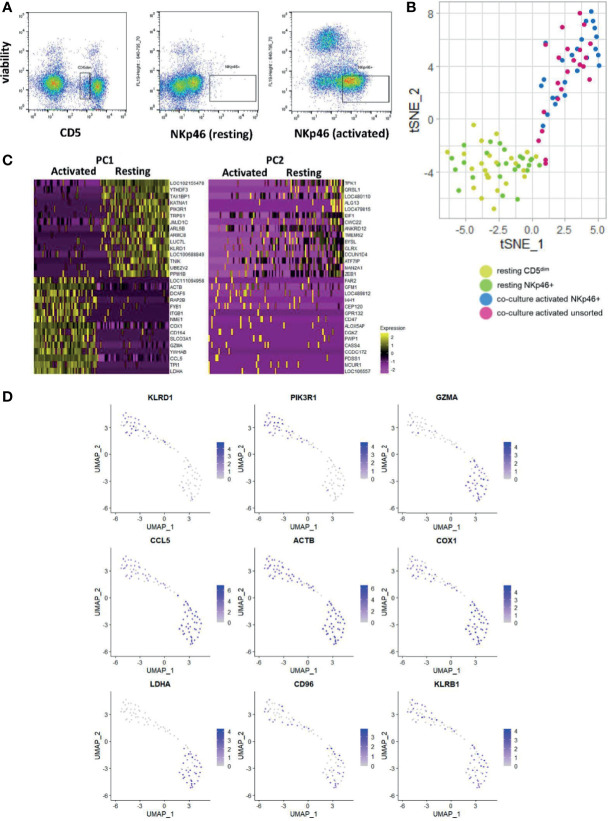
Single-cell analysis of activated CD5^dim^ and CD3-NKp46+ canine NK cells shows heterogeneity in time-to-response for NK cell activation. DGE following single-cell RNA sequencing of 4 populations: FACS-isolated resting CD5^dim^ and FACS-isolatedCD3-NKp46+ cells from healthy beagle PBMCs, co-culture activated CD3-NKp46+ cells and unsorted activated NK cells expanded from healthy beagle PBMCs using CD5 depletion. All sorting was completed using canine-specific monoclonal antibodies. ScRNASeq was performed using the plate-based 3’ UPX Transcriptome kit with 24 cells/group given the comparative rarity of the NKp46+ group. **(A)** Resting CD5^dim^, resting CD3-NKp46+, and activated CD3-NKp46+ populations are depicted as seen by flow cytometry during cell sorting procedure. Sorted populations lie within the designated gate. Note the markedly lower percentage of resting NKp46+ cells compared to the resting CD5^dim^ cells, highlighting that NKp46+ are a very small population of total PBMCs. **(B)** Dimensionality reduction using t-distributed stochastic neighbor embedding (t-SNE) plot of cells showing tighter clustering and high similarity among the activated CD3-NKp46+ and unsorted cell populations from a less dense distribution of the resting cell populations. Note, however, some activated, unsorted cells cluster with the resting CD5^dim^ and CD3-NKp46+ groups, suggesting that while all “activated” cells were exposed to K562/IL-2 co-culture for a uniform 14 days, biologically there exists heterogeneity in the time to response of single cells within the selected populations. **(C)** Heatmap depicting genes driving the variance for PC1 and PC2, representing how the gene expression of the combined resting and combined activated populations differ from one another. Resting CD5^dim^ and NKp46+ cells have higher expression of NKC gene KLRD1, and decreased expression of GZMA and CCL5 in comparison to the activated cell populations. **(D)** Panel of genes and corresponding expression in the single cell populations. Resting cells are in the top left portion of the graphs and activated cells are in the bottom right. Increasing gene expression is denoted by the color gradient. Note relatively constituitive expression of KLRB1, also known as NK1.1, which is a well-established phenotypic marker for murine NK cells and may represent a candidate in canines as well.

The utility of scRNAseq facilitates discovery of individual cell gene expression and allows for discovery of the gene expression patterns within specific cells, as opposed to composite comparisons. Therefore, we performed scRNAseq on resting FACS-isolated CD5^dim^ and NKp46+ cells, activated FACS-isolated NKp46+ cells and an unsorted activated population grown from CD5^dim^ cells (**Figure 5A**). [Canine NK cells downregulate CD5 expression following co-culture with K562clone9 and IL-2 (29)]. We hypothesized the groups of activated NK cells would exhibit variation in the precise level of gene expression on single-cell analysis. Our objective was to better characterize co-culture activated cells for translational purposes, such as autologous transfer, and identify potential NK cell subsets, known to exist in other mammals.

We performed t-stochastic neighbor embedding (tSNE) and observed the CD5^dim^ and CD3-NKp46+ resting populations formed a low-density grouping with mixed distribution of each cell type (**Figure 5B**). Following activation, the cells separate from the resting group to form a high-density group, again with no obvious clustering of cell type. However, select cells from the activated group cluster with the resting cells. Therefore, while all “activated” cells were exposed to K562/IL-2 co-culture for a uniform 14 days, biologically there exists heterogeneity in the time to response of single cells, and this may be explained by existence of cell subtypes.

All activated cells had higher expression of *GZMA, CCL5* and *LDHA*, while *PIK3R1* and *KLRD1* predominated in resting cells (**Figures 5C, D**). Of note, *KLRB1* was differentially expressed in the majority of resting and activated cells from both CD5^dim^ and CD3-NKp46+ populations. Also known as CD161/NK1.1, *KLRB1* is commonly used to identify NK cells in C57BL/6 and SJL mice, and is found on cytotoxic subpopulations of human NK cells (30, 31). Its expression is governed by the NKC (20). Therefore, *KLRB1*/CD161/NK1.1 may prove to be a feasible NK marker for canines and further highlights the importance of in-depth characterization of dog NK cells. In fact, KLRB1/NK1.1 presence has not previously been described in canines, underscoring the utility of gene expression profiling for identifying novel cross-species NK markers with translational relevance.

### 2.6 Stages of NK Cell Activation Develop in a Conserved Trajectory

**Figure 6:**
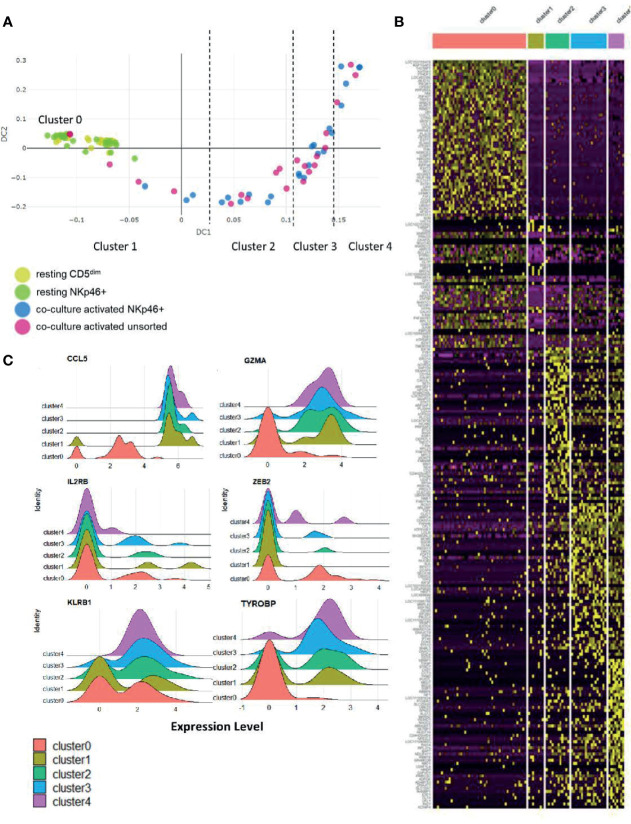
Stages of NK cell activation develop in a conserved trajectory. Diffusion mapping of cell populations illustrates a time-dependent diffusion process where the Euclidian distance between points approximates the diffusion distance. Populations used for cell sorting (resting CD5^dim^, resting CD3-NKp46+, activated CD3-NKp46+ and unsorted activated) are consistent with prior experimental conditions. **(A)** Here, we see an organized, uniform and quantifiable diffusion trajectory of activated CD3-NKp46+ and unsorted dog NK cells arising from resting CD5^dim^ cell populations, depicting heterogeneity in the biologic response to co-culture stimulation. Clusters along the trajectory have been divided by natural break points. **(B)** Visualization of gene expression changes between trajectories on heatmap. Note each cluster has a unique transcriptomic profile. **(C)** The top 10 differentially expressed genes for each cluster were collected. Genes that were noted to be differentially expressed between more than 1 cluster (suggesting stepwise changes throughout activation) were identified. Visualization of these genes are shown as ridge plots, which demonstrate changes in gene expression of these driving genes over time. Six genes, including canonical NK genes, had significant DGE between recurrent clusters: *CCL5, GZMA, IL2RB, ZEB2, KLRB1*, and *TYROBP* (Dap12).

To further explore the biology of time-to-response for activated NK cells described above, we then performed diffusion mapping. This revealed an organized trajectory of activated cells arising from a cluster of resting cells and demonstrates the differences in global gene expression at the single-cell level of activated NK cell populations. Some activated cells clustered with the resting phenotype while others demonstrated gene expression patterns consistent with progression to a more activated phenotype. Ultimately, we observed that the CD5^dim^ and CD3-NKp46+ cells followed a similar series of changes along an organized trajectory (**Figure 6A**).

To assess the genetic changes along this trajectory, we then divided the activated cells into clusters based on break points in diffusion distance with Cluster 1 being closest to the resting state and Cluster 4 being furthest from the resting state, with Cluster 0 representing the resting cells. Each cluster has a unique transcriptional profile (**Figure 6B** and **Supplemental Table 1**). Genes which changed expression significantly between multiple clusters (CCL5, GZMA, IL2RB, ZEB2, KLRB1 and TYROBP/DAP12) are shown in **Figure 6C**. Overall, these data demonstrate uniform, discreet changes in gene expression in canonical NK transcription factors that occur in stages during *ex vivo* activation. The subsets of NK cells along this trajectory possess different induced genes, and different repertoires of cell receptors. As repertoire diversity is known to occur in human NK cells (28), these data appear to be biologically significant to understand the transition of canine NK cells from a resting state to a co-culture activated state (a supraphysiologic process) and set the stage for studying NK cell maturation and activation *in vivo*, a physiologic process which has yet to be described in dogs.

### 2.7 IL-15 Treatment Distinctly Impacts Gene Expression of Canine NK Cells *In Vitro via* Alternative Activation Pathway

The effect of immune-stimulatory cytokines IL-2 and IL-15 on canine NK cell proliferation, morphology and cytotoxicity *in vitro* has been described, but limited gene expression analysis has been performed (16, 17). Work by our group has demonstrated a dose-dependent response of canine NK cells to rhIL-15 with regards to increased NK cytotoxicity (**Supplemental Figure 3**). To further understand the impact of activation and stimulation on dog NK gene expression, we next assessed DGE of dog CD5-depleted cells from PBMCs of healthy donors at rest, after treatment with rhIL-15, and again following K562 co-culture stimulation. We hypothesized that rhIL-15 would induce a unique transcriptional profile in NK cells when compared to no stimulation and co-culture.

**Figure 7:**
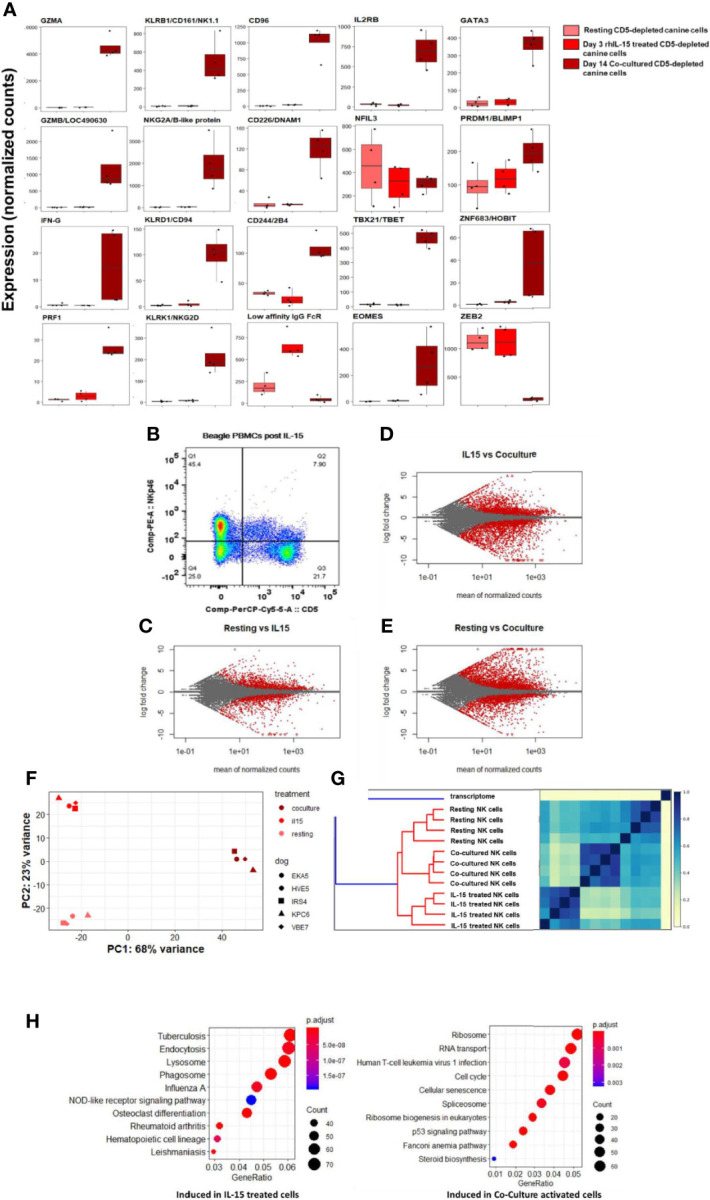
IL-15 treatment distinctly impacts gene expression of canine NK cells *in vitro via* alternative activation pathway. DGE between resting canine CD5-depleted cells, rhIL-15 treated CD5-depleted cells and cells activated *in vitro* by co-culture. PBMCs were obtained from healthy laboratory beagles, and NK cells were isolated using CD5-depletion *via* magnetic separation. The NK cells were then assessed at resting/steady-state, exposed to 3 days of 100 ng/mL recombinant human (rh) IL-15 or exposed to 14 days of co-culture with K562 cells and IL-2 (as before). **(A)** Individual normalized gene counts of canonical NK markers by treatment group. Within these classically NK-associated genes, variability is noted in the expression level between IL-15 treated and co-cultured cells. **(B)** Flow cytometry results of the CD5^dim^ and NKp46+ populations following exposure to IL-15. The expression of NKp46 increases significantly while the expression of CD5 is stable to decreased. These data are consistent with our co-culture data where CD5 expression goes down, either through cleavage or internalization, while NKp46 increases dramatically reaching approximately 100% by day 14 in co-culture conditions. **(C–E)** MA plot, mean of normalized counts as evaluated by log fold change, comparing differentially expressed genes (in red) between treatment conditions (resting, rhIL-15 treated and co-cultured), padj < 0.05. In total, **(B)** contains 1481 induced genes and 1362 repressed genes, **(C)** contains 2580 induced genes and 2948 repressed genes, and **(D)** contains 3305 induced genes and 2872 repressed genes which are differentially expressed between the cell populations. Note the highest amount of differentially expressed genes occurs between the resting and co-cultured conditions. **(F, G)** Two methods confirm distinct transcriptional profiles between resting, IL-15 treated and co-cultured conditions for CD5-depleted NK cells, supported by high similarity across four biological replicates: **(E)** similarity matrix as calculated by Jaccard Index of hash sketches of RNA sequencing data prior to alignment or normalization, **(F)** data visualization of principal component analysis. **(H)** Comparison of gene pathways induced following exposure to rhIL-15 and co-culture when using resting/steady-state conditions as referent. RhIL-15 treated cells appear to have high gene expression in pathways related to lysosome, endocytosis and phagosomes, as well as osteoclast-differentiation pathway related to inflammation. Co-cultured cells had higher gene expression in the cell cycle, p53 signaling, RNA transport, ribosome biogenesis and ribosome pathways. Of note, when co-culture conditions are compared to treatment with IL-15, the cellular senescence pathway is induced, potentially explaining why NK cells exposed to K562/IL-2 co-culture conditions become dysfunctional following adoptive transfer and fail to maintain an activated phenotype *in vivo*.

In comparison to co-cultured cells, rhIL-15 did not induce a significant gene expression change for most NK-related activating and maturation genes, but did induce high expression of CD16/IgG FcRIII (**Figure 7A**). Flow cytometry demonstrates the effect of *in vitro* incubation with IL-15 on the CD5^dim^ and NKp46+ populations with the expression of NKp46 increasing significantly while the expression of CD5 is stable to decreased (**Figure 7B** **).** MA plots showed thousands of differentially expressed genes between these three conditions, thus demonstrating that rhIL-15 stimulated dog NK cells have a unique transcriptional profile (**Figures 7C–E** and **Supplemental Figure 4**). Jaccard similarity and PCA showed reproducible independent signatures for each treatment group (**Figures 7F, G**).

To elucidate the unique biology of the rhIL-15 response, we then performed GO analysis. Importantly, there was no evidence of cellular senescence, which we observed in the co-culture activated cells (**Figure 7H**). In rhIL-15 treated cells, SPP1/OPN (osteopontin) was the most differentially expressed gene (average of normalized counts = 903.67, p_adj_ = 6.63e-69). Notably, OPN has been implicated as a key NK maturation factor and driver of NK-lineage development in mouse models, especially after IL-15 elaboration in the bone marrow microenvironment (**Figures 7E, H** and **Supplemental Figure 5**). We also observed that the lysosome pathway was highly induced following IL-15 stimulation, consistent with organelle turnover during autophagy. Thus, rhIL-15’s gene expression profile involves an alternative inflammatory pathway, regulation of dysfunctional components, and an absence of senescence when compared to co-cultured cells, overall demonstrating distinct immune responses when comparing IL-15 and co-culture stimulation.

### 2.8 NK Cells in Dogs With Naturally-Occurring Metastatic Osteosarcoma Differentially Respond to Inhaled rhIL-15

Access to companion animal dogs treated with inhaled rhIL-15 for metastatic OSA as part of novel comparative oncology clinical trial allowed us to compare the *in vitro* vs *in vivo* transcriptional profiles of rhIL-15 exposed dog NK cells. Among 7 dogs on a phase I investigational canine immunotherapy clinical trial available for analysis, we hypothesized that we would identify a distinct NK cell transcriptional profile between dogs responding and not-responding to treatment and that DGE of dogs responding to treatment would resemble those of cells treated with rhIL-15 *in vitro*.

**Figure 8:**
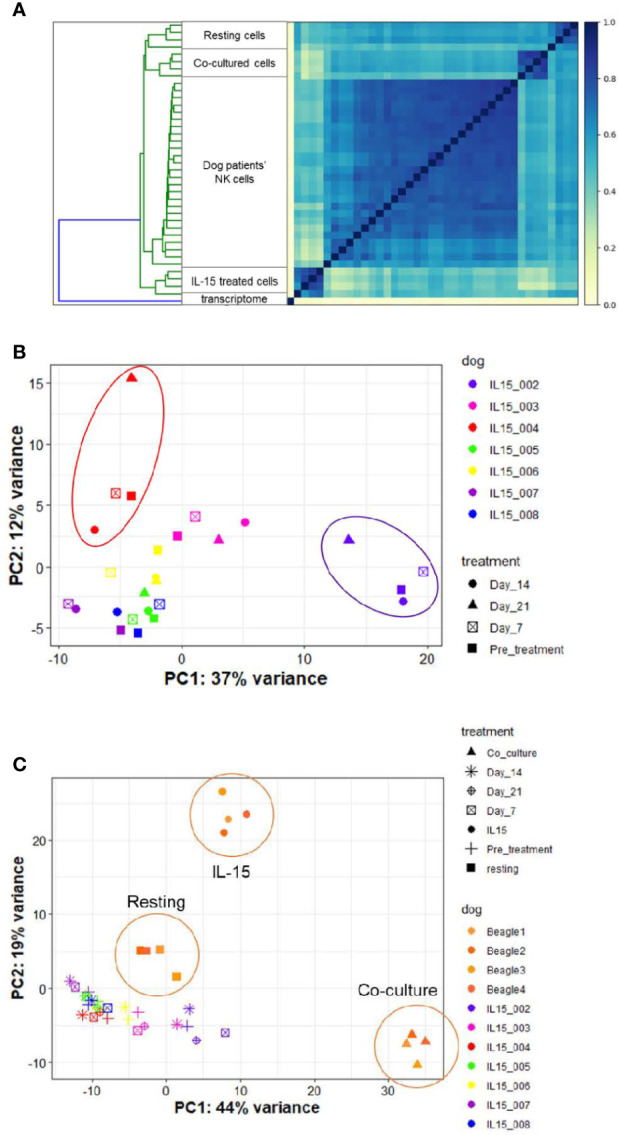
NK cells in dogs with naturally-occurring metastatic osteosarcoma differentially respond to inhaled rhIL-15. DGE between resting canine CD5-depleted cells, rhIL-15 treated cells and activated cells in vivo. The UC Davis Comparative Oncology Research Program is performing an IACUC-approved Phase 1 clinical trial evaluating inhaled rhIL-15 to treat pulmonary metastases in dogs with spontaneous osteosarcoma and melanoma. As such, we have been able to collect samples from dogs treated with a 14-day course of rhIL-15 in vivo and sequence CD5-depleted cells isolated from PBMCs. After owner consent, blood was obtained from trial dogs prior to treatment (pretreatment time point), and at 7, 14 and 21 days following treatment. **(A)** Similarity matrix of in vivo transcriptional profiles of canine cancer patients as compare to in vitro samples from healthy lab beagles. as calculated by Jaccard Index of hash sketches of RNA sequencing data prior to alignment or normalization. The dog patients form their own cluster on the matrix and bear the highest similarity to each other, irrespective of individual differences in dog breed or cancer type, when compared to in vitro samples obtained from healthy beagles. **(B, C)** PCA of in vitro and in vivo rhIL-15 treated canine peripheral blood samples. **(B)** PCA of in vivo samples only obtained from dog cancer patients on clinical trial of inhaled rhIL-15. **(C)** PCA of in vivo samples from canine cancer patients compared to resting and activated samples obtained from healthy lab beagles.

Endogenous NK cells from dog patients across time points bore the highest similarity to one another when compared to *in vitro* samples in either resting, IL-15 or activated conditions (**Figure 8A** and **Supplemental Figure 6** **).** On PCA, samples grouped by individual dog rather than by time point on therapy (**Figure 8B**). This suggests that clinical response may be dictated more by individual dog gene profile than by changes induced by treatment. Overall, the variance on PCA was driven by two dogs which appeared to experience clinical benefit from therapy, as one dog had prolonged stable disease and one dog had a partial response (IL15-002 and IL15-004, respectively). When we compared the gene profiles of circulating NK cells from cancer-bearing dogs receiving inhaled rhIL-15 to the NK cells from healthy beagles treated with rhIL-15 *in vitro*, we observed that the majority of variation between dog patients was obscured (**Figure 8C**). In fact, DGE profiles from dog OSA patients most closely resembled those of resting NK cells, even for dogs responding to treatment. These results highlight the differential responses of immune subsets like NK cells between *in vivo* and *in vitro* conditions, likely reflecting key differences in pharmacokinetics, metabolism and bio-energetics, as well as interactions with other cellular immune and non-immune constituents which all contribute to immune phenotype and function *in vivo*.

### 2.9 Cross-Species Analysis Shows Greater Similarity of Canine NK Cells to Humans Than to Mice

Critical differences exist between human and mouse NK cells, including but not limited to cell surface markers, patterns of lymph node trafficking, differentiation stages, and mechanisms of MHC class recognition. Consequently, it is imperative to characterize the comparative immunogenomics for NK cells across the spectrum of mice, dogs, and humans to bridge cross-species studies and advance clinical translation. To this end, we evaluated differential gene expression of phenotypically comparable CD3-NKp46+ cell populations from humans, dogs and mice in the resting state and after comparable stimulation using rhIL-2 for 7 days. Given the potential for species-specific responses to the human K562 cell line, co-culture with K562 cells were not used for this experiment.

Such approaches have previously been limited due to a reliance on microarray panels of predicted genes in targeted regions of the transcriptome without providing a global picture of cellular transcription. Leveraging modern advances in orthologous gene predictions, we identified all 1:1 orthologous genes between mice, dogs and humans and constructed a novel orthologous transcriptome of over 7000 genes to serve as a reference comparative transcriptome between species.

**Figure 9:**
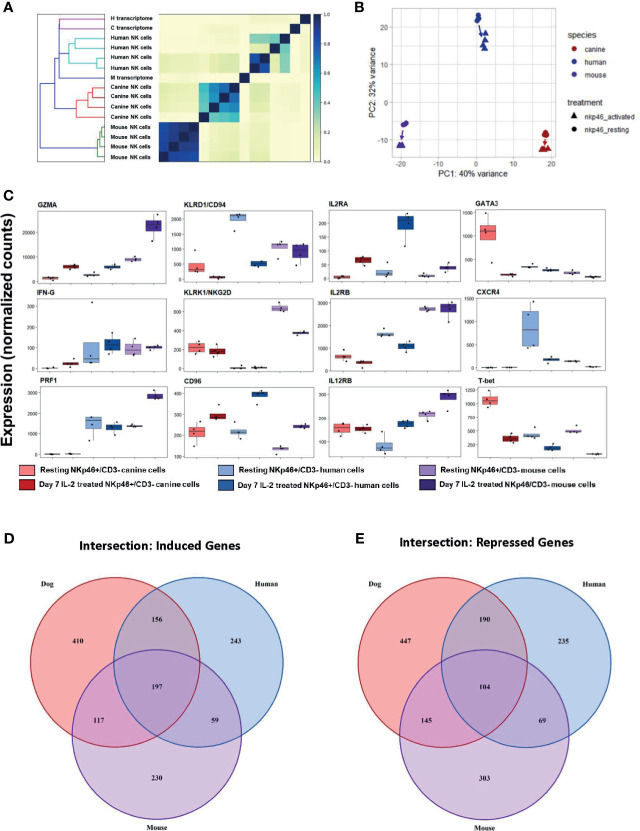
Cross-species analysis shows greater similarity of canine NK cells to humans than to mice. Cross species analysis of CD3-NKp46+ cell populations for humans, canines and mouse populations. All species were sequenced in the resting and “activated” conditions. All human, canine and mouse CD3-NKp46+ cells were exposed to 1000IU/ml rhIL-2 for 7 days). In order to conduct this experiment, a cross-species reference transcriptome was required. Tables of orthologous genes between species were constructed preserving 1:1:1 orthologous genes for all species. The final annotated transcriptome was comprised of over 7,000 genes shared between mice, canines and humans. **(A)** Similarity matrix as calculated by Jaccard Index of hash sketches of RNA sequencing data prior to alignment or normalization. On dendrogram (left), mice cluster as an outgroup when compared to canines and humans. Note Jaccard Index approaches 1 for mouse samples, signifying a nearly identical state. This speaks to the high degree of inbreeding for mice. Conversely, the canine replicates remain heterogeneous, despite all samples originating from farm-bred inbred beagles. Thus, while dogs are inbred with respect to specific breed, it is not to the extreme of laboratory mice. **(B)** PCA for all three species. Difference in mouse and canine NK cells drive the variance for PC1, whereas mouse and canines are equidistant from humans and drive variance for PC2. **(C)** Individual normalized orthologous gene counts of canonical NK markers by species and treatment group: dog activated and resting cells, human activated and resting cells, and mouse resting an activated cells. **(D, E)** Venn diagram depicts the intersection of **(D)** induced and **(E)** repressed genes between the resting and activation conditions for NK cells across mouse, dog and human using our standardized reference transcriptome. Dogs, humans and mice share 197 induced genes following co-culture. In addition to this, dogs and humans share another 156 induces genes, which humans and mice share only 59 more genes. With respect to repressed genes, all three species share 104 similarly repressed genes. Once again, dogs and humans share an additional 190 repressed genes, while humans and mice share only 69. Based on total number of induced and repressed genes, dogs NK cells share significantly more common genes with human NK cells than the mouse model.

We first calculated Jaccard similarity, a critical step since it is based on hash sketches from pre-processed reads and does not rely on alignment to a transcriptome (32). Jaccard similarity with dendrogram demonstrates mice as an outgroup compared to dog and human (**Figure 9A**). Mouse samples were nearly identical whereas canine cells had variability comparable to healthy human, despite dog samples all originating from farm-bred beagles. These data confirm the extreme degree of inbreeding in laboratory mice while suggesting more NK genetic diversity/variation in farm-bred beagles than anticipated. PCA was performed with orthologous gene counts, and demonstrates, canine and mouse NK cells were equidistant from human cells, as shown in **Figure 9B**, PC2. Hierarchical similarity clustering by heatmap shows increased similarity between dog and human gene expression, corroborating the findings of Jaccard similarity (**Supplemental Figure 7**). Taken together, these data demonstrate the global transcriptional profile of canine NK cells has greater similarity to human NK cells than to mouse.

While canine-human similarity in the global transcriptome is evident, we also sought to compare the expression of specific genes. Expression of NKC-associated genes between resting and activated cells demonstrated significant heterogeneity across species (**Figure 9C**). We then quantified the number of shared induced and shared repressed genes between species. Importantly, the intersections of both induced and repressed genes shared between dog and human were higher than between human and mouse or dog and mouse, respectively (**Figures 9D, E**). Dogs, humans and mice share 197 induced genes following co-culture. In addition to this, dogs and humans share another 156 induces genes, which humans and mice share only 59 more genes. With respect to repressed genes, all three species share 104 similarly repressed genes. Once again, dogs and humans share an additional 190 repressed genes, while humans and mice share only 69. Taken together, this data demonstrates dogs and humans share greater numbers of induced and repressed genes involved in NK function compared to mice and are thus transcriptionally more similar.

## 3 Discussion

Here we present the first analysis of the canine NK cell transcriptome using high-throughput RNA sequencing techniques and bioinformatic analysis. This fills an important gap in the literature, as these cells are poorly defined but of great potential to advance the field of NK biology and cancer immunotherapy. The lack of available species-specific antibodies has previously hindered efforts to study canine NK cells. Importantly, RNASeq provides the ability to design and perform hypothesis-driven experiments using in-depth analysis of transcriptional profiles unique to dogs and customized to their genetic makeup.

Our first objective was to define the gene expression profiles of putative NK populations based on the two currently utilized principal phenotypic surface markers for canine NK cells: CD5^dim^ and NKp46+. The results of our analysis demonstrate that CD5^dim^ is not specific for identifying circulating NK cells (based on DGE analysis of canonical NK markers), whereas NKp46 shows high concordance for canonical NK markers identified in mouse and human NK cells. Of note, however, NKp46 expression in dogs may not identify all NK subtypes in the steady state. Resting CD5-depleted (isolated by magnetic separation) and resting CD3-NKp46+ populations (isolated by cell sorting) were highly distinct in terms of transcriptional profiles on bulk RNAseq, whereas FACS-isolated resting CD5^dim^ and NKp46+ cells showed some overlap on scRNAseq PCA. Interestingly, resting CD5^dim^ outliers drove the variance indicative of a more heterogeneous population.

Ultimately, examination of the differentially expressed genes between the CD5-depleted, CD5^dim^ and NKp46+ canine NK cells demonstrates genes commonly associated with immune cells of different lineages in the CD5-depleted and CD5^dim^ populations. Thus, identification of cells by a low density of the CD5 surface receptor, whether by magnetic separation or flow sorting, is unlikely to yield a specific dog NK population. For this reason, while CD3-NKp46+ delineation may not identify all dog NK cells, transcriptional evidence clearly points to this phenotype as a higher fidelity for a homogenous population with high expression of canonical NK cell genes.

Remarkably, regardless of which phenotypic surface marker was expressed on the starting population, the transcriptional profiles converged to a statistically indistinguishable degree of similarity after 14 days of co-culture with K562 feeder cells and low-dose IL-2 (22, 29). These data also suggest that NKp46 also may be a marker for NK cell maturation in dogs, given the impressive increase in expression after co-culture with near 100% expression by day 14. Diffusion mapping further elucidated time-dependent genetic changes throughout the activation process, which offer many avenues for future study of canine NK cell development, function and terminal differentiation. All populations expressed KLRB1/NK1.1, which appears to represent a novel constitutive NK marker in dogs throughout expansion.

Interestingly, we observed distinct patterns of differential gene expression following stimulation of canine NK cells with rhIL-15. These gene expression data are consistent with studies in humans as well as mice which have shown that IL-15 preferentially affects NK cell survival and activation rather than proliferation and expansion (16). Given these prior observations, it is fitting that our data demonstrate distinct transcriptional profiles for cells exposed to rhIL-15 and co-culture. A persistent hurdle for adoptive transfer of NK cells in cancer patients is depressed cytotoxicity and survival of exogenously-expanded NK cells after adoptive transfer (14, 33). It has been previously postulated that co-cultured cells, while highly active *in vitro*, are simultaneously developing a dysfunctional phenotype which is unmasked following adoptive transfer. This notion is supported by our finding of a highly induced cellular senescence pathway in the co-cultured cells. However, it is important to note the duration of exposure to co-culture is 14 days, whereas rhIL-15 exposure is 3 days, and duration of stimulation may be a significant factor contributing to senescence with co-culture.

Access to specimens from canine cancer patients receiving immunotherapy further allowed us to test our hypotheses regarding differences in transcriptional profiles *in vivo* versus *in vitro* using high impact patient-derived specimens. Our findings speak to the importance of *in vivo* models, as transcriptional profiles changed markedly between the *in vitro* and *in vivo* conditions. Admittedly, there are many variables which could be contributing to these differences. First, our *in vitro* samples were obtained from healthy dogs with no history of cancer, although we also analyzed pre-treatment samples from our cancer patient cohort as relevant controls. Cancer, particularly when metastatic, is a systemic disease that causes immune system dysfunction and thus changes in differential gene expression. Secondly, *in vitro* NK cells were exposed to higher doses of rhIL-15 than the canine patients in the clinical trial. It is also known in other mammals that tissue-resident NK cells have important differences (24). NK cells responding to the rhIL-15 in the lungs of dogs may have a different transcriptional profile than the NK cells remaining in the periphery, and this is a key question for future studies.

The clinical trial of inhaled IL-15 is not powered at this point to detect efficacy and therefore statements regarding significant differences in transcriptional profiles between clinically responding and non-responding dogs are premature. However, with seven subjects, our study does demonstrate that transcriptional profiles grouped by individual dog under hierarchical similarity clustering and not duration of treatment. This suggests that any response to cytokine treatment is likely to be driven by an individual subject’s NK cell transcriptional profile at baseline and not by cumulative dose. Therefore, it may be possible in the future to develop a panel of pre-treatment genes to predict response to IL-15 and other cytokine therapies, an important step towards selecting biomarker-driven therapies for immune therapies.

Finally, we are the first to do comparative transcriptomics of canine NK cells in comparison to mouse and human NK cells. Our method involved the creation of a novel cross-species transcriptome, preserving every gene where there was a 1:1:1 ortholog between our three species of interest and therefore yielding a more complete comparison. Previous transcriptomic cross-species analyses between mice and humans have relied on microarray data which are subject to bias *via* omission of genes outside those hypothesized to be of interest (34). Our method is important as it provides a comprehensive analysis of the dog NK transcriptome in relation to human and mouse (C57BL/6) NK cells. Although mouse splenocytes were used to isolate NK cells, each species’ cells were positively selected using flow cytometry and sorted for a uniform CD3-NKp46+ phenotype. IL-2 was used in place of K562 cells in order to avoid species-specific reactions to expansion. In summary, our findings can help facilitate translation of preclinical findings discovered in mice, as canine clinical trials can speed assessment of efficacy and toxicity signals and thereby de-risk innovative NK immunotherapy approaches before testing in humans, in so doing benefitting the health of both species.

It is important to acknowledge the limitations of our study. First, the dog reference transcriptome is markedly less complete than those for mice and humans. Although we provide the first in-depth analysis of the dog NK transcriptome with novel insights into activation and phenotype, additional NK genes of interest remain undefined in the dog transcriptome (e.g. KIR and Ly49) and will yield even greater information when annotation is improved. Secondly, proper identification of pure NK cell populations remains an issue in canines (as in other species) and can be considered a potential limitation of our work. However, an important goal of this study was to ameliorate this limitation and improve characterization of dog NK cells for cross-species studies. As discussed previously, CD5 depletion by magnetic beads has been associated with the presence of B cells and macrophages in the initial product, and we addressed this limitation by using cell sorting for pure CD5^dim^ populations in both scRNAseq and bulk RNASeq experiments where we again observed transcriptional heterogeneity within purified CD5^dim^ populations.

In summary, our work provides a detailed atlas of transcriptional changes of canine NK cells under a variety of conditions and highlights many future directions for investigation. Most notably, we describe the expression of genes which are translated to cell surface proteins that have not been described previously in canines, including KRLB1 (NK1.1), CD96, KLRF1, and KLRK1 (NKG2D), among others. Data such as these can now be used to advance the field of comparative oncology, optimize clinical applications of NK immunotherapy and improve the health of humans and companion canines. Ultimately, our cross-species data also demonstrate the similarities of dog NK cells to human NK cells and underline the potential for canine studies to shed insight on key questions in NK biology such as memory, tissue residence, and cancer surveillance.

## 4 Methods

### 4.1 Processing of Canine NK Cells

Canine whole blood was obtained from healthy female beagles (Ridglan Farms, Mount Horeb, Wisconsin) aged 2 – 8 years old or dog patients enrolled on an IACUC and Clinical Trials Review Board-approved clinical trial (Inhaled IL-15 Immunotherapy for Treatment of Lung Metastases, Protocol #20179). PBMCs were collected using a density gradient (Lymphocyte Separation Medium, Corning Life Sciences). Red blood cells were eliminated by incubation with RBC lysis buffer for five minutes at 4°C. Cells were then washed with PBS and utilized for subset isolation. CD5^dim^/neg cells were isolated using the Easy Sep PE Positive Selection Kit (Stem Cell Technologies, Vancouver, BC) and PE-conjugated anti-canine CD5 (Invitrogen, clone YKIX322.3). Studies were in compliance with ethical regulations of AALAC and The Clinical Trials Review Board.

### 4.2 Processing of Human NK Cells

Human leukocyte reduction system chambers were obtained from healthy, unidentified volunteers age 20 – 65 (Blood Source, Sacramento, CA) and were exempt from IRB approval. Blood samples were diluted 1:1 in PBS, followed by collection of peripheral blood mononuclear cells (PBMCs) using a density gradient (Lymphocyte Separation Medium, Corning Life Sciences). Red blood cells were eliminated using RBC lysis buffer for five minutes at 4°C. Cells were then washed with PBS and utilized for NK cell isolation. Human NK cells were isolated from PBMCs using the Rosette Sep Human NK Isolation Kit according to the manufacturer’s specifications (Stem Cell Technologies, Vancouver, BC).

### 4.3 Mice

Female 10-12 week old C57BL/6J mice were purchased from Jackson Laboratories (West Sacramento, CA). Mice were housed in AALAC-accredited animal facilities at the University of California, Davis (UC Davis) under specific-pathogen-free conditions. Protocols were approved by the UC Davis IACUC Protocol #20707, and studies were in compliance with ethical regulations and humane endpoints.

### 4.4 Processing of Mouse Tissue

Following approved euthanasia technique, mouse spleens from 12-week old C57BL/6J were collected and processed to generate single cell suspensions for analysis or culture. Mouse spleens were mechanically dissociated in PBS. Tissue was then strained through a 70 µm filter and cells were treated with RBC lysis buffer for 5 minutes (BioLegend #420301). Cells were then washed with PBS, strained, and resuspended in PBS. Mouse NK isolation was done *via* the MagniSort Mouse NK cell enrichment Kit (Thermo Fisher #8804-6828).

### 4.5 *Ex Vivo* NK Cell Activation and Expansion of Human and Canine NK Cells

The NK fraction were grown in complete RPMI, resuspended at 5 × 10^6^ cells/mL with irradiated K562 human feeder cells transduced with 4-1BBL (CD137L) and membrane-bound rh-IL21 (K562C9IL21, kind gift of Dr. Dean Lee, Nationwide Children’s Hospital, Columbus, Ohio) at a ratio of 1:2 (NK:feeder cells) supplemented with 100 IU/mL rh-IL2 (25–28). The parental K562 cell line was originally obtained from American Type Culture Collection (ATCC) prior to engineering of transgene expression (29). 100 IU/mL recombinant human IL-2 (NCI, Frederick, MD) was added every 2–3 days while NK cells were in culture. Every 7 days, cells were counted and resuspended at a concentration 250,000 NK cells/mL with fresh K562C9IL21 at a ratio of 1:1, as previously described (29).

### *4.6 Ex Vivo* NK Cell Activation and Expansion of Murine NK Cells

Mouse splenocytes were cultured in complete RPMI supplemented with recombinant human IL-2 at 1000 IU/mL for 7 days. Half-volume media changes were performed every 2-3 days to refresh media and replace IL-2. All cells were cultured in incubators at 37°C at 5% CO2 in complete RPMI media (RPMI 1640 media (Invitrogen Life Technologies) supplemented with 10% Nu-Serum (Corning Life Sciences), 2mM L-glutamine (Glutamax^®^, Gibco), 1% non-essential amino acids (Gibco), 5x10-5M β-mercaptoethanol (MP Biomedicals), 1mM Hepes buffer (Corning Life Sciences), 1mM sodium pyruvate (Gibco), and 1% penicillin/streptomycin (Corning Life Sciences).

### 4.7 Cross Species *In Vitro* Studies

Cytokine-activated NK cells were generated from human and canine peripheral blood and mouse splenocytes. Human NK cells, canine PBMCs, and mouse splenocytes were each cultured in complete RPMI supplemented with recombinant human IL-2 at 1000 IU/mL for 7 days. Half-volume media changes were performed every 2-3 days to refresh media and replace IL-2. At time of collection, cells were vigorously pipetted and mechanically lifted from the plate surface to ensure collection of adherent cells. Cells were then washed in PBS and utilized for cell sorting. All cells were cultured in incubators at 37oC at 5% CO2 in complete RPMI media (RPMI 1640 media (Invitrogen Life Technologies) supplemented with 10% Nu-Serum (Corning Life Sciences), 2mM L-glutamine (Glutamax^®^, Gibco), 1% non-essential amino acids (Gibco), 5x10-5M b-mercaptoethanol (MP Biomedicals), 1mM Hepes buffer (Corning Life Sciences), 1mM sodium pyruvate (Gibco), and 1% penicillin/streptomycin (Corning Life Sciences).

### 4.8 Antibody Staining and Cell Sorting

Antibody staining and flow cytometry. Cells were prepared in a single cell suspension. Cells were washed with PBS, incubated with the species-specific Fc receptor blocking solution (Human TruStain FcX, BioLegend #422302; Canine Fc Receptor Binding Inhibitor, Invitrogen #14-9162-42; Mouse TruStain FcX, BioLegend #101320), then stained with the following fluorochrome-conjugated monoclonal antibodies:

#### 4.8.1 For Human Cells

CD3-FITC (clone HIT3a, BioLegend #300306), NKp46-BV605 (clone 9E2, BioLegend #331926), Live/dead staining performed using Fixable Viability Dye 780 (eBioscience #65-0865-14).

#### 4.8.2 For Canine Cells

CD3-FITC (clone CA17.2A12, BioRad #MCA1774F), NKp46-PE (clone 48A, kind gift of Dr. Dean Lee), CD5 on PerCP-eFluor 710 (clone YKIX322.3) Thermo Fisher #46-5050-42, Live/dead staining performed using Fixable Viability Dye 780 (eBioscience #65-0865-14).

#### 4.8.3 For Mouse Cells

CD3-BV785 (clone 17A2, BioLegend #100232), NKp46-PerCP-Cy5.5 (clone 29A1.4, BioLegend #560800), Live/dead staining performed using Fixable Viability Dye 780 (eBioscience #65-0865-14).

### 4.9 Flow Cytometry and Cell sorting

PBMCs from dog blood were stained with monoclonal antibodies to identify canine CD5 and NKp46 expression for flow cytometric analysis and cell sorting using a Beckman Coulter Astrios EQ 18 color cell sorter. Several precautions were taken to configure the Astrios to ensure sample quality for genomic analysis. Sample carryover and contamination were reduced by flushing the fluid lines with diethylpyrocarbonate (DEPC)- treated water prior to sample introduction, sample processing time was reduced by using a 70um nozzle at 60 psi fluidic pressure and sample vitality was protected during plate sorts by chilling the unsorted sample reservoir and collection vessels. The sorter’s electronic circuitry and cell sorting gates were adjusted to exclude small sub-cellular debris and larger multi-cell aggregates based on 488nm laser light scattering. Dead cells that may exhibit non-specific staining in channels used to detect canine cells were excluded based on bright positive fluorescence using Near Infrared Live/Dead viability stain. Candidate single, live, canine cells were identified and gated to select only anti-canine CD5^dim^ and NKp46^bright^ fluorescent signals. The sorter’s deposition unit was configured either for single cell deposition into 96 well PCR-type plates and the sorter’s droplet deflection was modified to allow for vertical particle deposition. Cells were sorted directly into 96-well plates containing QIAseq lysis buffer and RNAse inhibitor. A plate map was prepared to reflect the 24 wells allotted for each treatment condition.

### 4.10 RNA Sequencing

RNA from isolated NK cells for each sample was extracted using RNeasy Mini kits (Qiagen). Total RNA was submitted to the UC Davis Genome Center for quality assessment, library preparation and sequencing. Gene expression profiling was carried out using a 3’-Tag-RNA-Seq protocol (35). 4 biologic replicates were used for each set of experimental conditions for bulk RNAseq, and 24 biologic replicates (wells) for each single cell experimental condition. Barcoded sequencing libraries were prepared using the QuantSeq FWD kit (Lexogen, Vienna, Austria) for multiplexed sequencing according to the recommendations of the manufacturer using both, the UDI-adapter and UMI Second-Strand Synthesis modules (Lexogen). The fragment size distribution of the libraries was verified *via* micro-capillary gel electrophoresis on a LabChip GX system (PerkinElmer, Waltham, MA). The libraries were quantified by fluorometry on a Qubit fluorometer (LifeTechnologies, Carlsbad, CA), and pooled in equimolar ratios. The library pool was quantified *via* qPCR with a Kapa Library Quant kit (KapaBiosystems/Roche, Basel, Switzerland) on a QuantStudio 5 system (Applied Biosystems, Foster City, CA). Up to forty-eight libraries were sequenced per lane on a HiSeq 4000 sequencer (Illumina, San Diego, CA) with single-end 100 bp reads.

### 4.11 Single Cell RNA Sequencing

Single-cell gene expression studies were carried out with a 3’ RNA-seq protocol employing the QIAseq UPX 3’ Transcriptome Kit (Qiagen, Hilden, Germany) according to the recommendations of the manufacturer.

Reverse transcription was primed with poly-T tailed oligonucleotides (Oiagen) containing also unique molecular identifier sequences (UMIs), well-specific barcode sequences, and Illumina adapter sequences in the presence of template switching oligos. After heat inactivation of the reverse transcriptase, up to 96 reactions were combined and purified with magnetic SPRI beads (Qiagen). A qPCR amplification was carried out on an aliquot of the cDNA pool to determine the optimal cycle number for the PCR amplification of the cDNA pool. After PCR amplification of the remainder of the cDNA, the PCR products were bead purified, enzymatically fragmented, A-tailed and ligated to barcoded Illumina compatible adapters (Qiagen) adding a plate-specific index. The fragment size distribution of the libraries was verified *via* micro-capillary gel electrophoresis on a LabChip GX system (PerkinElmer, Waltham, MA). Sequencing libraries were quantified by fluorometry on a Qubit fluorometer (LifeTechnologies, Carlsbad, CA), and pooled in equimolar ratios. The library pool was quantified *via* qPCR with a Kapa Library Quant kit (Kapa Biosystems/Roche, Basel,Switzerland) on a QuantStudio 5 system (Applied Biosystems, Foster City, CA). Libraries representing multiple 96-well plates were sequenced on a NextSeq 500 sequencer mid-output run (Illumina, San Diego, CA).

### 4.12 Data Analysis

Computational resources were provided by JetStream/XSEDE as a startup allocation (36, 37).

Raw fastq files generated by 3’ Tag-Seq underwent preprocessing *via* a reproducible pipeline using *snakemake* (38). Samples were concatenated, followed by trimming of the first 12 bases and quality trimming using *bbduk_qc* (39). Non-ribosomal RNA was identified and selected for using *bbduk_find_ribo* (39). Reads were then indexed to the reference transcriptome for canines (CanFam3.1) (40), humans (GRCh38.p13) (41) or mice (GRCm38.p6) (42) and counts generated with *salmon (* 43).

Hash sketches from processed RNA sequences were generated by *sourmash* and assessed for similarity (44). Count files generated by *salmon* were then read into R using the *tximport* package (45). Differential gene expression analysis was done using the *DESeq2* package for R, and differentially expressed genes identified based on a negative binomial distribution, with maximum likelihood estimation, and using the Bayes theorem to guide quantitative movement for each gene based on observed counts. The Benjamini-Hochberg procedure used to control false discovery rate (46). FDR threshold was based on a padj < 0.01 and a log2FC cut off of 0.4. Tables of orthologous genes were constructed using OMA Browser’s Genome Pair View (47). Cross-species comparison was conducted with the *UpSetR* package (48). For gene ontology analysis based on KEGG pathways, the R packages *clusterProfiler* and *enrichplot* were used (49–51).

Single cell fastq files were processed using *UMI tools* to extract individual and cell barcodes and *STAR* for mapping and alignment (52–54). The R package *Rtnse* was used to create the t-distributed Stochastic Neighbor-Embedding plot, *destiny* was used to create the diffusion map, and *Seurat* used for differential expression (55–57).

Detailed code for all analyses can be found on GitHub: https://github.com/alicia-gingrich

FASTQ files and quantified gene counts for RNA sequencing and single-cell sequencing are available from the Sequence Read Archive under accession number BioProject Number PRJNA682461.

## Data Availability Statement

The datasets presented in this study can be found in online repositories. The names of the repository/repositories and accession number(s) can be found below: https://www.ncbi.nlm.nih.gov/, PRJNA682461.

## Ethics Statement

The animal study was reviewed and approved by UC Davis IACUC and Clinical Trials Review Board-approved clinical trial: Inhaled IL-15 Immunotherapy for Treatment of Lung Metastases, Protocol #20179. Written informed consent was obtained from the owners for the participation of their animals in this study.

## Author Contributions

AG designed the experiments, performed bioinformatics analysis and cell sorting, produced the figures and wrote the manuscript. TR assisted in design of the experiments, bioinformatics analysis and statistical considerations. SJ, DY, MY, AR, IA, UN, RB, and KS performed cell isolation and staining from mouse, dog and human cells; performed RNA extraction and managed samples from clinical trial patients. WM provided immunology expertise and critical review of the manuscript. RR provided veterinary expertise, funding and critical review of the manuscript. CB provided bioinformatics expertise, funding and critical review of the manuscript. RC designed the experiments, provided oncologic and immunology expertise, funding and critical review of the manuscript. All authors contributed to the article and approved the submitted version.

## Funding

This work was supported in part by National Institutes of Health/National Cancer Institute grant U01 CA224166-01 (RC, RR, CB) and R03CA252793 (RC).

## Conflict of Interest

The authors declare that the research was conducted in the absence of any commercial or financial relationships that could be construed as a potential conflict of interest.

## Publisher’s Note

All claims expressed in this article are solely those of the authors and do not necessarily represent those of their affiliated organizations, or those of the publisher, the editors and the reviewers. Any product that may be evaluated in this article, or claim that may be made by its manufacturer, is not guaranteed or endorsed by the publisher.
