# Supplementary material for: Comparative Immunogenomics of Canine Natural Killer Cells as Immunotherapy Target
Source: Front Immunol. 2021 Sep 14;12:670309. doi: 10.3389/fimmu.2021.670309 (PMC8476892; doi:10.3389/fimmu.2021.670309)
Supplement: Supplementary file 1 [file DataSheet_1.pdf]

Supplemental Figure 1:

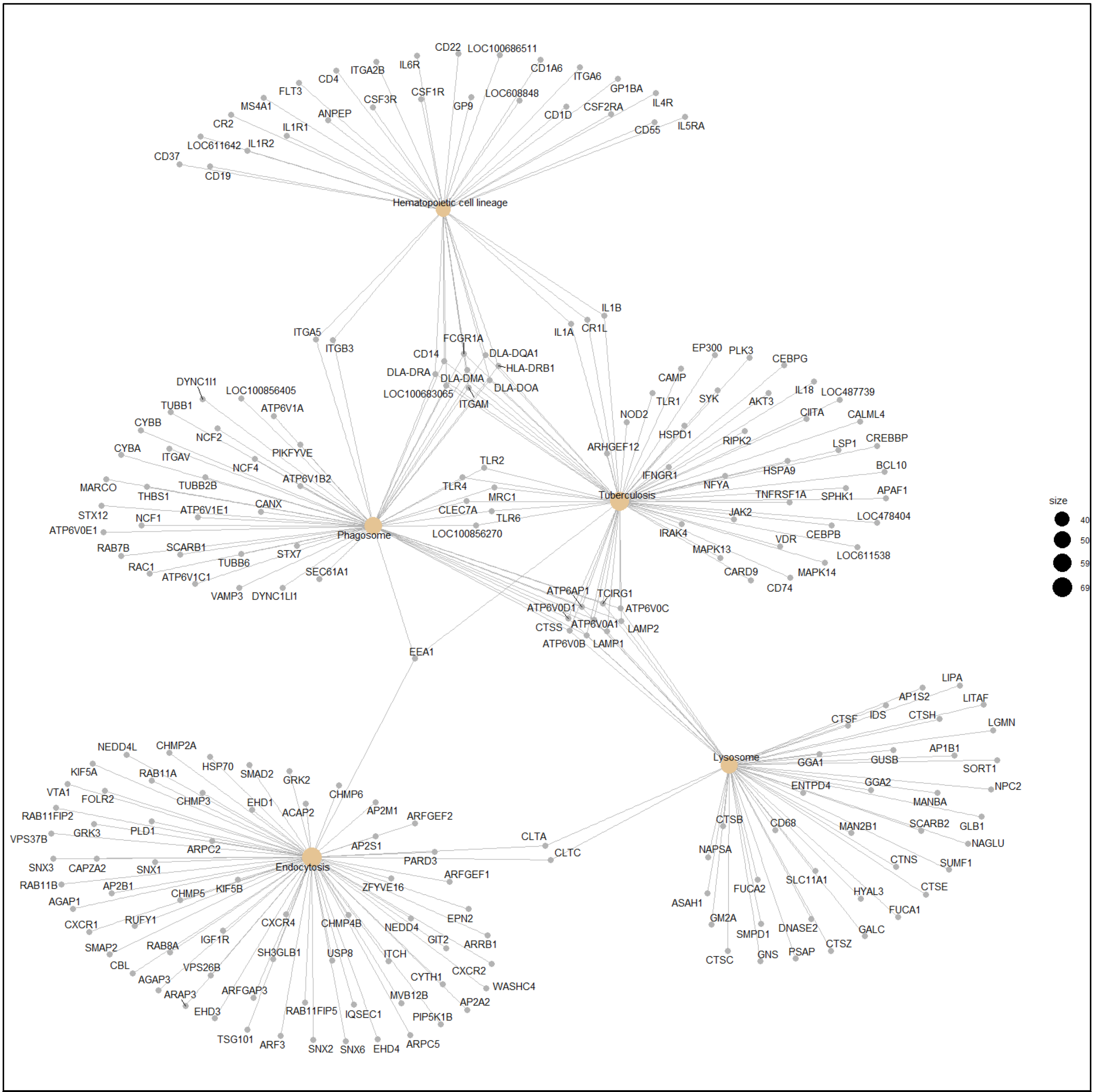

Supplemental Figure 1a: Gene networks induced resting (steady-state) CD5-depleted canine cells when compared to resting NKp46+ canine cells, as described by KEGG pathways.

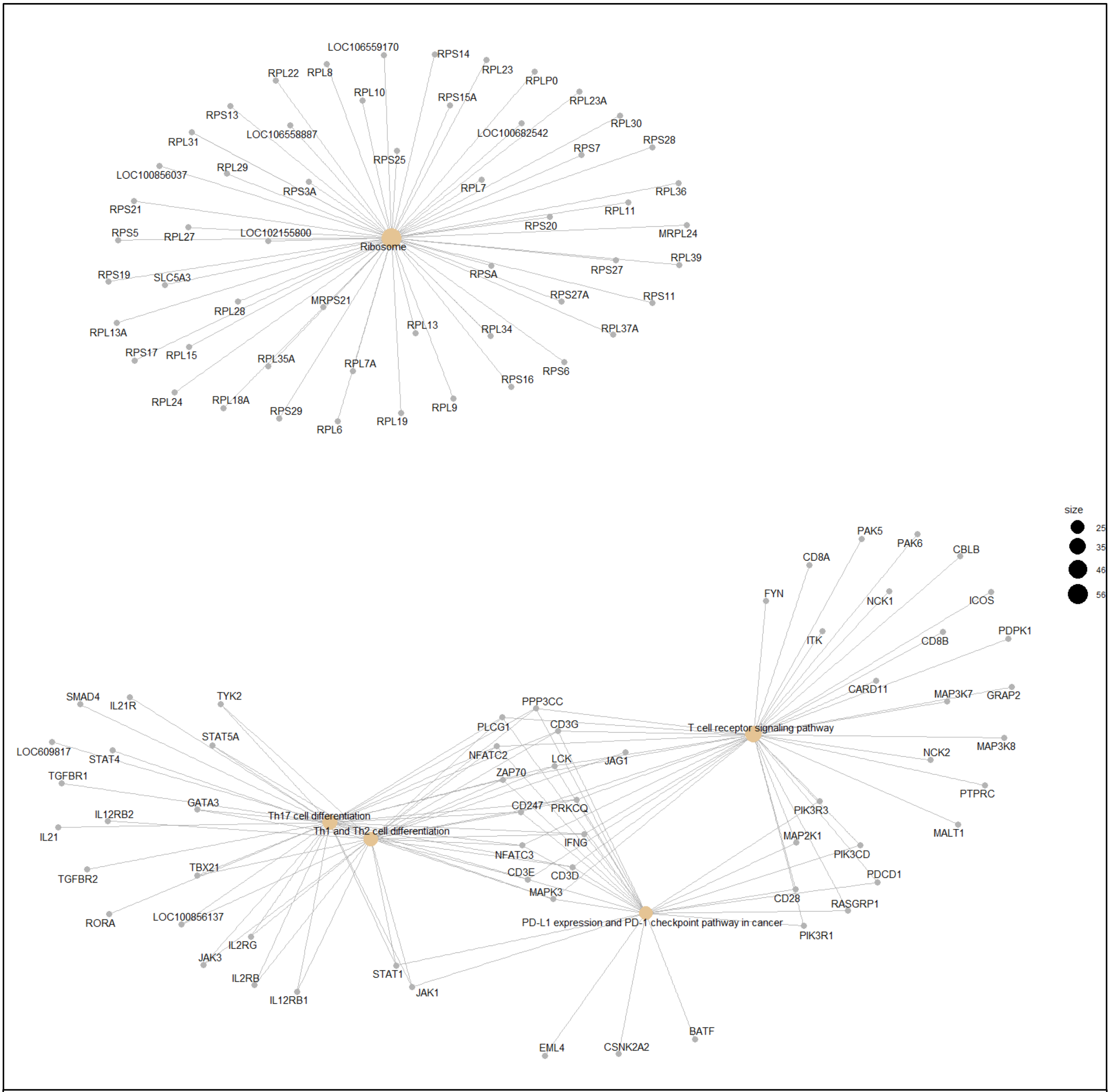

Supplemental Figure 1b: Gene networks induced resting (steady-state) NKp46+ canine cells when compared to resting CD5-depleted canine cells, as described by KEGG pathways.

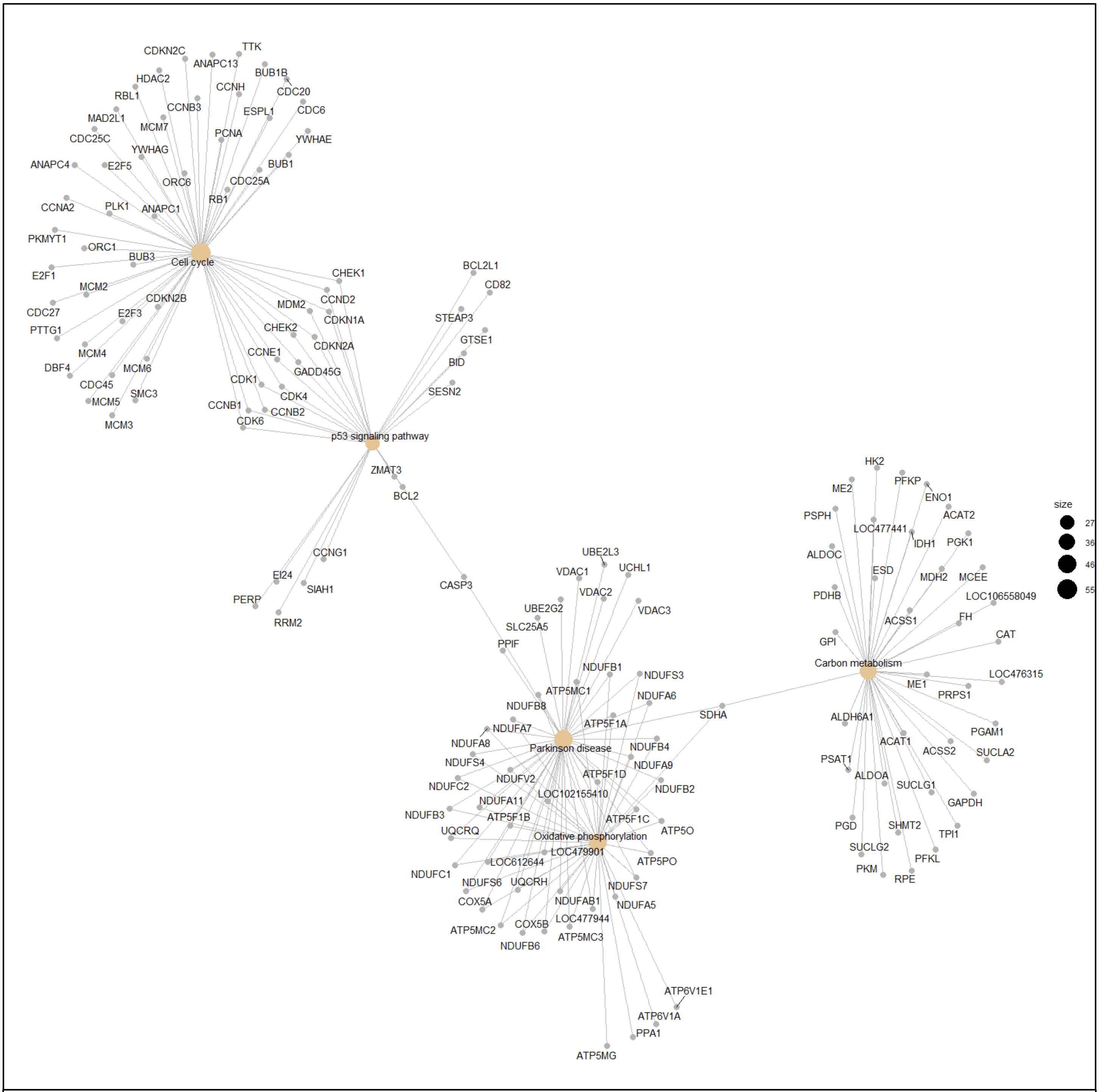

Supplemental Figure 1c: Gene networks induced K562 co-culture activated NKp46+ canine cells when compared to resting (steady state) NKp46+ canine cells, as described by KEGG pathways.

Supplemental Table 1: Differentially expressed genes in clusters when compared to all clusters

Cluster	Gene	Average Log Fold Change	P Value
Cluster 0	LOC102155478 (uncharacterized)	2.377821754	8.05E-16
	RAP1GAP2	3.107314801	9.83E-16
	TAX1BP1	2.426421391	1.54E-13
	KATNA1	2.47978088	5.08E-12
	PIK3R1	2.355959366	1.07E-10
	TRPS1	2.175365029	1.04E-09
	ARMC8	2.176310626	1.89E-09
	KLRD1	2.370081661	2.01E-09
	QKI	2.403063516	4.73E-09
	FAF2	2.232475934	1.02E-06
Cluster 1	TARBP1	1.749144511	0.000366
	TXN2	1.429730698	0.000366
	HYPK	2.294247949	0.004865
	GALK2	1.584255988	0.004949
Cluster 2	EIF3K	1.512559589	5.06E-06
	FYB1	1.842829426	9.91E-06
	ACVR2A	1.981605457	2.87E-05
	SAP130	1.772527242	3.21E-05
	CD164	1.530598487	6.28E-05
	ARFGEF1	2.048222307	0.000225
	PRPSAP2	1.577435382	0.003367
	BAG6	1.544299076	0.003604
	CEP57L1	1.511963008	0.003855
	CD2	1.518381513	0.005585
Cluster 3	ARPC4	1.43672353	2.70E-06
	ATP6V0E1	1.277343661	5.92E-06
	LDLR	1.406794809	8.43E-06
	ORC5	1.26958187	3.74E-05
	SLK	1.392533877	9.45E-05
	SEC61B	1.429049602	0.000179
	TPP2	1.560444954	0.000307
	DENR	1.254823433	0.001184
	DNAJC19	1.484034035	0.003353
	IFT46	1.336303318	0.003896
Cluster 4	TXNIP	1.46236634	1.67E-08
	PUS7L	1.455034562	2.41E-07
	SSR1	1.328042991	2.75E-07
	PTGER3	1.585455886	4.19E-06
	SLC25A20	1.651784137	4.79E-06
	SHOC2	1.320615235	2.08E-05
	NFE2L2	1.257790953	6.02E-05
	AMD1	1.543755092	0.00028
	GAPVD1	1.26519052	0.000774
	RANBP1	1.304488852	0.00269

Supplemental Figure 2:

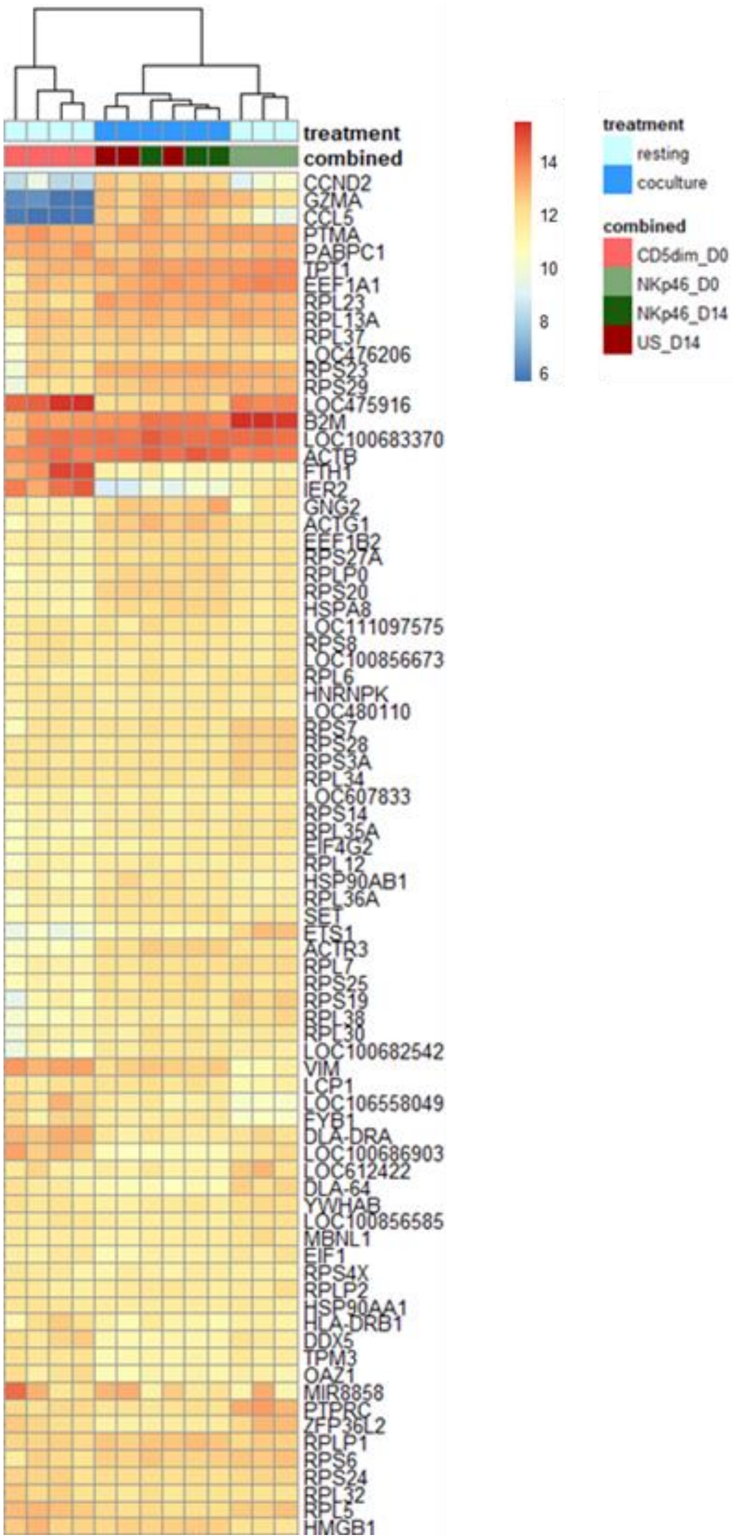

Supplemental Figure 2: Heatmap of the top 100 differentially expressed genes for resting CD5-depleted cells, resting NKp46+ cells, co-culture activated NKp46+ cells and activated unsorted cells. Note the high degree of similarity between the two activated populations in contrast with the different transcriptional profiles of the starting populations.

Supplemental Figure 3:

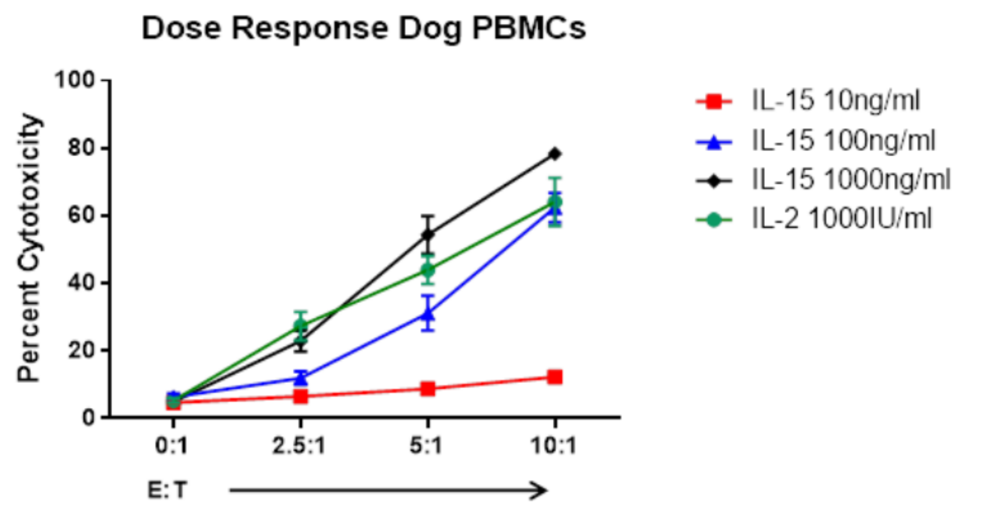

Supplemental Figure 3: Results from 24-hour killing assay in which day 0 canine PBMCs were exposed to rhIL-15 for an incubation time of 64 hours. Cytotoxicity was measured against the canine thyroid adenocarcinoma cell (CTAC) cell line, a classic target to assess canine NK cell function). Note cytotoxicity increases at the 100ng/mL dose, which was the *in vitro* dose used for the canine NK cells prior to sequencing in this study.

Supplemental Figure 4:

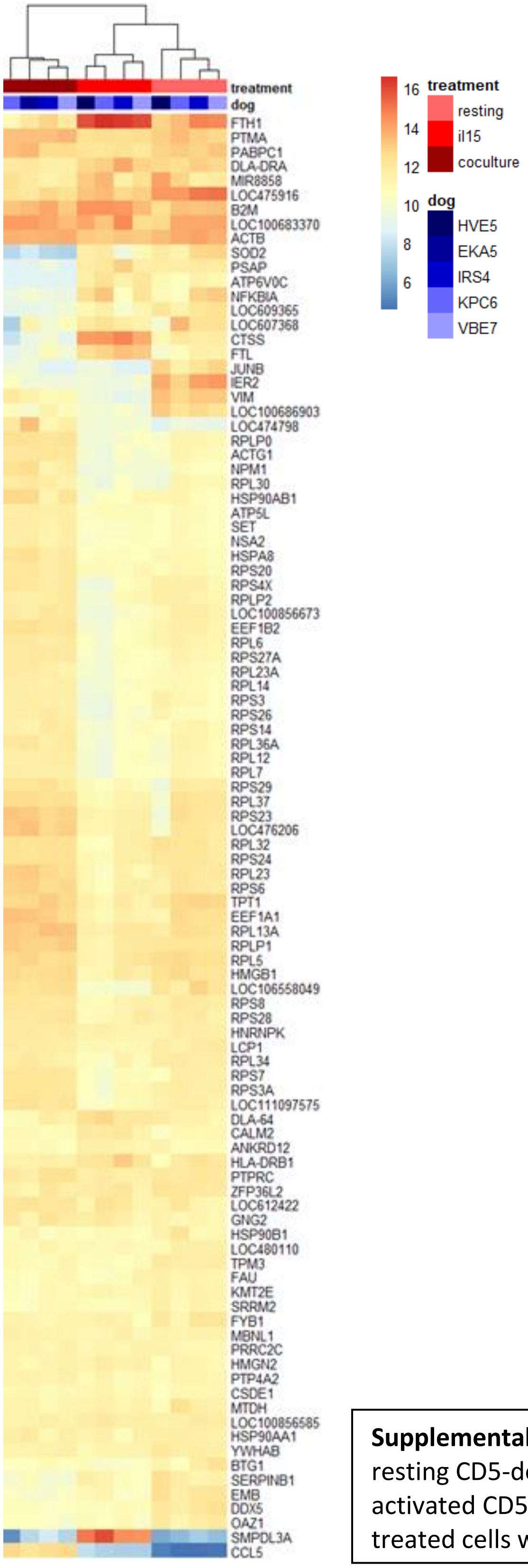

Supplemental Figure 4: Heatmap of the top 100 differentially expressed genes for resting CD5-depleted cells, IL-15 treated CD5-depleted cells, and co-culture activated CD5-depleted cells. Note the unique transcriptional profile of the IL-15 treated cells with respect to the co-culture activated cells.

Supplemental Figure 5:

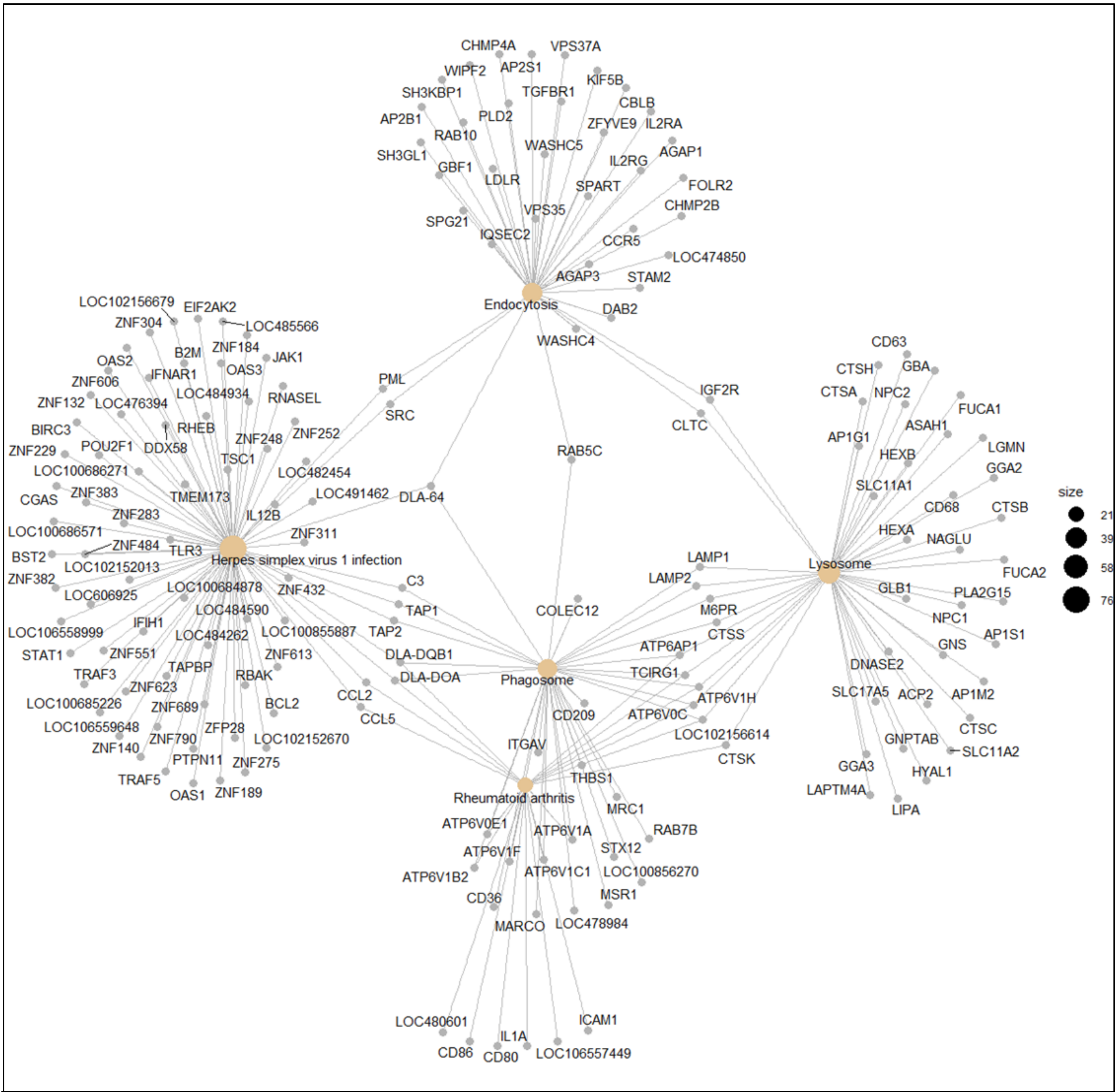

Supplemental Figure 5a: Gene networks induced in rhIL-15 treated canine NK cells when compared to resting (not treated) canine NK cells, as described by KEGG pathways.

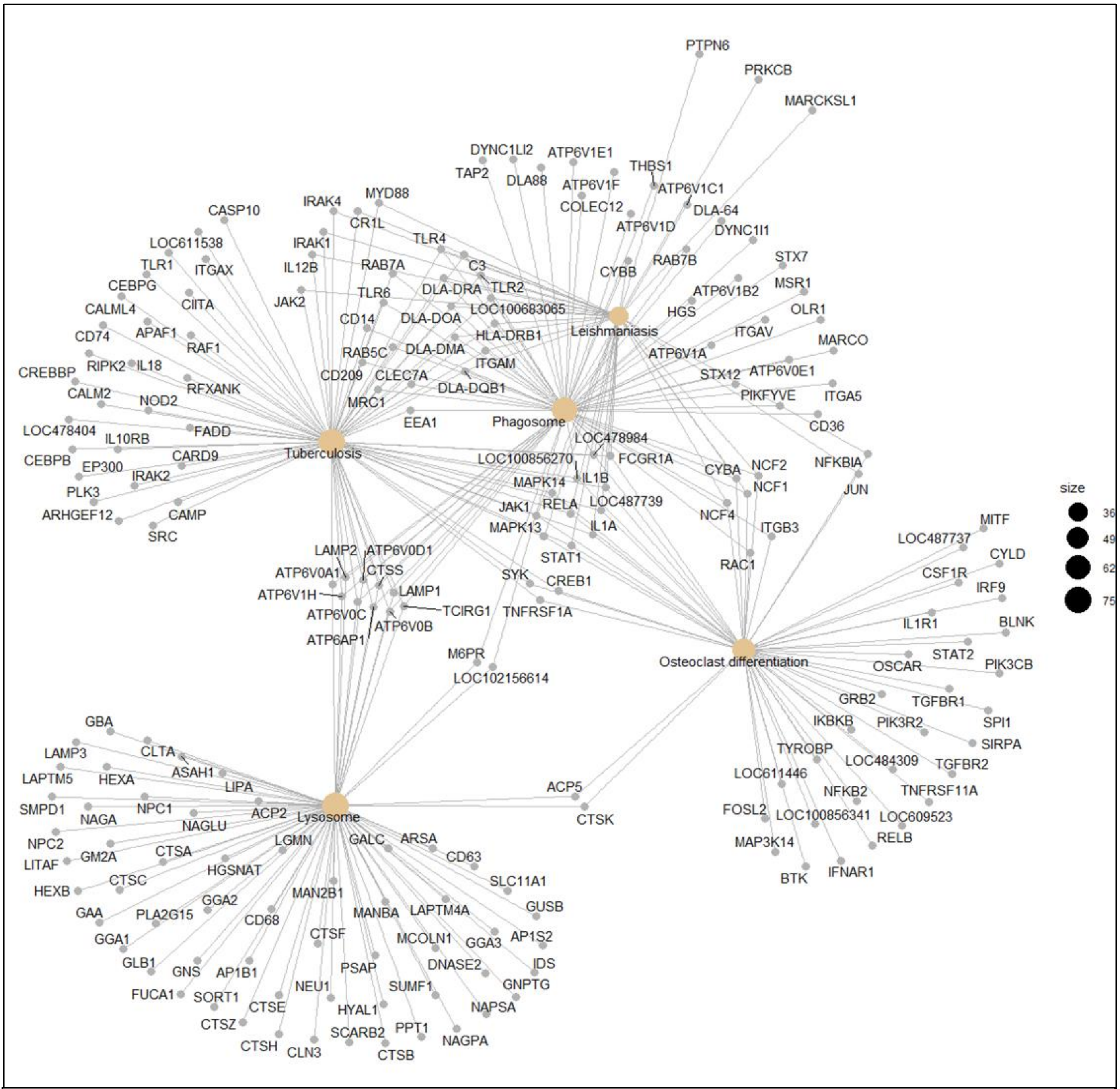

Supplemental Figure 5b: Gene networks induced in rhIL-15 treated canine NK cells when compared to co-cultured (K562 and IL-2 exposed) canine NK cells, as described by KEGG pathways.

Supplemental Figure 6:

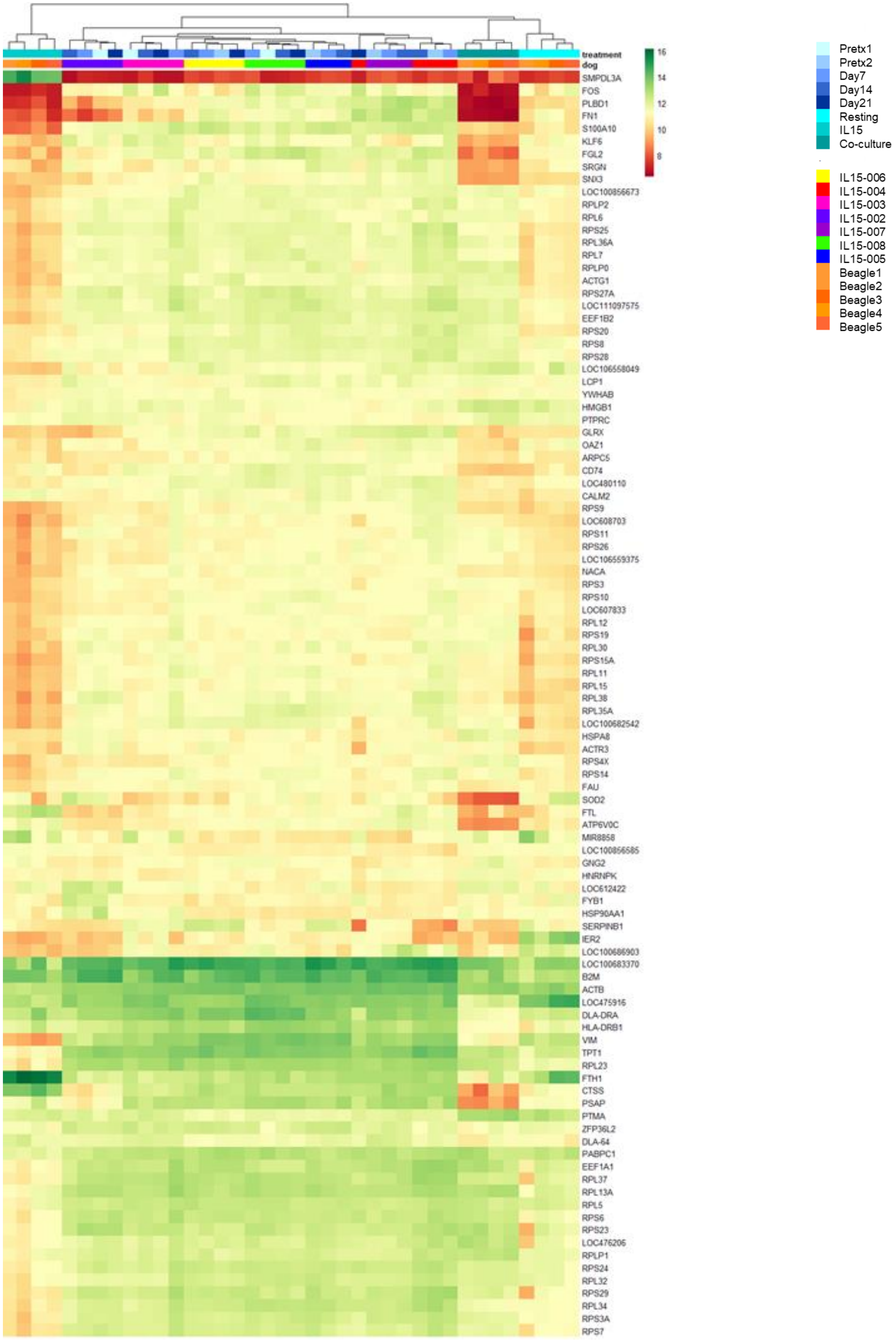

Supplemental Figure 6: Heatmap of the top 100 differentially expressed NK genes for the canine clinical trial patients in conjunction with the NK cells from healthy beagles treated from resting conditions or treated with IL-15 or co-culture in vitro.

Supplemental Figure 7:

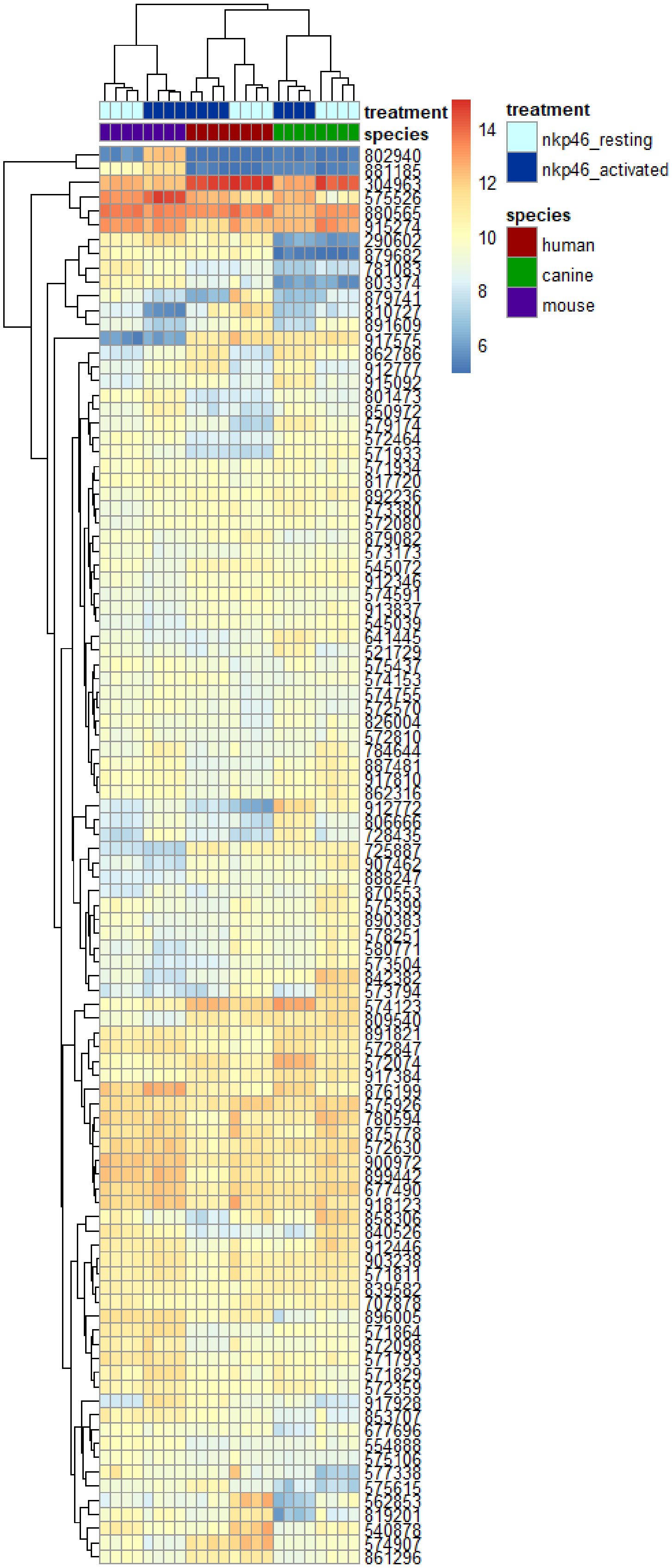

EntrezID	Gene symbol
802940	SPP1
881185	CCR2
304963	B2M
575526	GZMA
880565	TPT1
915274	RPS23
290602	PRF1
879682	FCER1G
781083	SERINC3
803374	CD52

Supplemental Figure 7: Cross-species heatmap for gene expression of resting and activated NK cells for mouse, dog and human. Transcriptomes were merged on EntrezID given software parameters; Table gives top 10 EntrezID to gene symbol conversion and corresponds to right axis of heatmap.
